# Curing of Cocoa Beans: Fine-Scale Monitoring of the Starter Cultures Applied and Metabolomics of the Fermentation and Drying Steps

**DOI:** 10.3389/fmicb.2020.616875

**Published:** 2021-01-11

**Authors:** Cristian Díaz-Muñoz, Dario Van de Voorde, Andrea Comasio, Marko Verce, Carlos Eduardo Hernandez, Stefan Weckx, Luc De Vuyst

**Affiliations:** ^1^Research Group of Industrial Microbiology and Food Biotechnology, Faculty of Sciences and Bioengineering Sciences, Vrije Universiteit Brussel, Brussels, Belgium; ^2^Laboratorio de Calidad e Innovación Agroalimentaria, Escuela de Ciencias Agrarias, Universidad Nacional de Costa Rica, Heredia, Costa Rica

**Keywords:** cocoa fermentation, cocoa bean drying, starter culture, amplicon sequence variant, metabolomics, yeasts, lactic acid bacteria, acetic acid bacteria

## Abstract

Starter culture-initiated cocoa fermentation processes can be applied to improve the quality of cured cocoa beans. However, an accurate monitoring of the microbial strains inoculated in fresh cocoa pulp-bean mass to assess their contribution to the cocoa bean curing process is still lacking. In the present study, eight different cocoa fermentation processes were carried out with Trinitario cocoa in vessels in Costa Rica to assess the contribution of two candidate yeast starter culture strains, namely *Saccharomyces cerevisiae* IMDO 050523 and *Pichia kudriavzevii* IMDO 020508, inoculated in combination with *Limosilactobacillus fermentum* IMDO 0611222 and *Acetobacter pasteurianus* IMDO 0506386. A multiphasic approach, consisting of culture-dependent selective plating and incubation, rRNA-PCR-DGGE community profiling of agar plate washes, and culture-independent high-throughput amplicon sequencing, combined with a metabolite target analysis of non-volatile and volatile organic compounds (VOCs), was performed on samples from the fermentation and/or drying steps. The different starter culture mixtures applied effectively steered the cocoa fermentation processes performed. Moreover, the use of an amplicon sequence variant (ASV) approach, aligning these ASVs to the whole-genome sequences of the inoculated strains, allowed the monitoring of these inoculated strains and their differentiation from very closely related variants naturally present in the background or spontaneous fermentation processes. Further, traits such as malolactic fermentation during the fermentation step and acetoin and tetramethylpyrazine formation during the drying step could be unraveled. Finally, the yeast strains inoculated influenced the substrate consumption and metabolite production during all starter culture-initiated fermentation processes. This had an impact on the VOC profiles of the cured cocoa beans. Whereas the *P. kudriavzevii* strain produced a wide range of VOCs in the cocoa pulp, the *S. cerevisiae* strain mostly influenced the VOC composition of the cured cocoa beans.

## Introduction

Cocoa fermentation and drying are key steps in the curing of cocoa beans to make them ready for the production of chocolate (Schwan and Wheals, 2004; Saltini et al., 2013; De Vuyst and Weckx, 2016; Pereira et al., 2016; Ozturk and Young, 2017; Castro-Alayo et al., 2019; Figueroa-Hernández et al., 2019; De Vuyst and Leroy, 2020; Santander Muñoz et al., 2020). The microbial communities involved in cocoa fermentation processes are mainly composed of yeasts (in particular *Candida*, *Hanseniaspora*, *Saccharomyces*, and *Pichia* species), lactic acid bacteria (LAB; in particular *Lactiplantibacillus* and *Limosilactobacillus* species), and acetic acid bacteria (AAB; in particular *Acetobacter* species). However, other microbial groups, such as enterobacteria, *Bacillus* species, and/or filamentous fungi, may also appear.

Traditionally, cocoa fermentation processes are spontaneous, uncontrolled, on-farm processes (Schwan and Wheals, 2004; De Vuyst and Weckx, 2016; Pereira et al., 2016; Ozturk and Young, 2017; Figueroa-Hernández et al., 2019; De Vuyst and Leroy, 2020; Santander Muñoz et al., 2020). They usually consist of two phases. The anaerobic phase is dominated by yeasts and LAB as the first microbial groups that are metabolically active. During this phase, carbohydrates (mainly sucrose, glucose, and fructose) and citrate present in the cocoa pulp-bean mass are consumed, pectin is degraded (liquifying the pulp, causing a drainage or sweatings), and carbon dioxide, ethanol, glycerol, lactate, mannitol, and flavor compounds (e.g., pyruvate metabolites and amino acid conversion products) are produced. During the following aerobic phase, the microbial group of AAB oxidizes the ethanol produced by the yeasts into acetate. These microbial activities cause an increase of the temperature of the fermenting cocoa pulp-bean mass, because of the exothermic effects of fermentation and, in particular, the ethanol oxidation and further overoxidation of acetate. An initial slight pH increase is caused mainly by the consumption of citrate during the anaerobic phase, followed by a slight pH decrease because of mainly acetate production during the aerobic phase. Acetate overoxidation may again cause a pH increase upon prolonged fermentation. The effect of the temperature and pH shift, together with the flow of ethanol and acetate from the pulp into the beans, is responsible for the killing of the seed embryo, the destruction of the seed structure, and the activation of endogenous enzymes that give the final flavor and color of the cured cocoa beans, in turn influencing the flavor characteristics of the chocolates produced from the concomitant roasted beans (Schwan and Wheals, 2004; Afoakwa et al., 2008; Saltini et al., 2013; Kongor et al., 2016; De Vuyst and Leroy, 2020; Santander Muñoz et al., 2020).

By the addition of a starter culture, the whole cocoa fermentation process can be better controlled, reaching a more uniform quality of cured cocoa beans (Lefeber et al., 2012; De Vuyst and Weckx, 2016; De Vuyst and Leroy, 2020). In the past decade, the use of starter cultures for cocoa fermentation has been focused on enhanced pulp drainage, faster fermentation of the cocoa pulp-bean mass, and improved quality of the cured and roasted cocoa beans and the chocolates produced thereof. They enabled competitiveness with or inhibition of the background microbiota and improvement of the quality and sensory profiles of the end-products (Saltini et al., 2013; De Vuyst and Weckx, 2016; Pereira et al., 2016; Ozturk and Young, 2017; Castro-Alayo et al., 2019; Figueroa-Hernández et al., 2019; Mota-Gutierrez et al., 2019; De Vuyst and Leroy, 2020; Santander Muñoz et al., 2020). Therefore, several studies have been performed to select strains of dominant and/or beneficial yeast and bacterial species occurring during spontaneous cocoa fermentation processes to elaborate appropriate inoculation cocktails (Lefeber et al., 2010, 2011; Pereira et al., 2012; Moens et al., 2014; Samagaci et al., 2014a,b; Meersman et al., 2015; Visintin et al., 2016; Koffi et al., 2018). Successful controlled cocoa fermentation processes have been carried out with mixed-strain starter cultures of *Saccharomyces cerevisiae*, *Limosilactobacillus fermentum* (formerly known as *Lactobacillus fermentum*) and/or *Lactiplantibacillus plantarum* (formerly known as *Lactobacillus plantarum*), and *Acetobacter pasteurianus* and/or *Acetobacter aceti* (Schwan, 1998; Lefeber et al., 2012; Sandhya et al., 2016; Moreira et al., 2017; Ho et al., 2018). However, in general, the often minor impact of most starter culture-initiated cocoa fermentation processes on the quality of the final chocolates (Leal et al., 2008; Lefeber et al., 2012; Crafack et al., 2013, 2014; Batista et al., 2015; Menezes et al., 2016; Sandhya et al., 2016; Moreira et al., 2017; Visintin et al., 2017; Ho et al., 2018; Mota-Gutierrez et al., 2018; Assi-Clair et al., 2019) and doubts about the necessity of LAB and AAB for successful cocoa fermentation (Ho et al., 2015, 2018; Meersman et al., 2015; Moreira et al., 2017; John et al., 2020) have moved the interest in the application of dedicated bacterial strains toward the development of performant yeast starter cultures, whether or not in combination with LAB and/or AAB strains (Pereira et al., 2012; Meersman et al., 2015, 2016; Ho et al., 2018). The underperformance of the former starter cultures used regarding chocolate flavor may be ascribed to the low-cell-density inocula applied or the production of off-flavors by the background microbiota in the case that the added starter cultures were not competitive enough to prevail. Therefore, further careful monitoring of the microbial community dynamics by means of state-of-the-art techniques, as part of a multiphasic approach, is still necessary to confirm the prevalence of desirable yeast and bacterial species throughout the fermentation process after inoculation of appropriate strains, and to help to elucidate their contribution to the final flavor profiles of the cured cocoa beans and concomitant chocolates.

Whereas the species diversity of LAB and AAB is rather limited, that of the yeasts is more diverse (Daniel et al., 2009; Papalexandratou and De Vuyst, 2011; Maura et al., 2016; De Vuyst and Leroy, 2020). Nevertheless, as mentioned above, yeasts are key microorganisms without which cocoa fermentation cannot develop properly, hence resulting in less cured beans in their absence (Lefeber et al., 2012; Ho et al., 2014, 2015, 2018). A number of researchers have indicated diverse yeast strains to be part of an inoculation cocktail or even as the sole inoculated microorganisms for cocoa fermentation processes based on their ethanol production and tolerance (Daniel et al., 2009; Lefeber et al., 2012; Pereira et al., 2012; Visintin et al., 2016; Koffi et al., 2018). Moreover, extensive microbial community dynamic studies have shown a pivotal role for *Hanseniaspora*, *Pichia*, and/or *Saccharomyces* species (Lagunes Gálvez et al., 2007; Daniel et al., 2009; Papalexandratou and De Vuyst, 2011; Meersman et al., 2013; Papalexandratou et al., 2013; De Vuyst and Weckx, 2016; Koné et al., 2016; Maura et al., 2016; Ho et al., 2018; De Vuyst and Leroy, 2020). Indeed, *Hanseniaspora* species (in particular *Hanseniaspora opuntiae*) often appear at early stages of the cocoa fermentation process, which later disappear in favor of more ethanol-tolerant *Saccharomyces* species (in particular *S. cerevisiae*) or *Pichia* species (in particular *Pichia kudriavzevii*), once high ethanol concentrations occur after approximately 48 h of fermentation. Furthermore, the selection of yeast strains that produce a wide range of different flavor compounds, which may be of influence on the flavor of the final chocolates, is of increasing importance (Crafack et al., 2013, 2014; Ramos et al., 2014; Meersman et al., 2016; Menezes et al., 2016; Pereira et al., 2017; Assi-Clair et al., 2019; Castro-Alayo et al., 2019; De Vuyst and Leroy, 2020). Indeed, during cocoa fermentation, a vast number of volatile organic compounds (VOCs), next to non-volatile ones, is produced, many of which are the result of the yeast metabolism (Schwan and Wheals, 2004; Aculey et al., 2010; Rodriguez-Campos et al., 2011; Ho et al., 2014; Ramos et al., 2014; Koné et al., 2016; Menezes et al., 2016; De Vuyst and Leroy, 2020). In particular, higher aldehydes, higher alcohols, certain organic acids, and esters are produced mainly by yeasts. Yet, many of these yeast flavor compounds are associated with a high intraspecies diversity, indicating the importance of a rational selection of candidate yeast strains for starter culture applications (Meersman et al., 2016; Pereira et al., 2017). In particular, *P. kudriavzevii* seems to be a contributor of certain flavor compounds, such as higher aldehydes and esters, albeit strain-dependent (Pereira et al., 2017). It has been suggested as potential starter culture by several authors (Pereira et al., 2012, 2017; Ho et al., 2018; Ooi et al., 2020). However, to have an impact on the flavor quality of the cured cocoa beans and, possibly, of the chocolates produced therefrom, the VOCs produced in the cocoa pulp-bean mass have to diffuse into the beans during the fermentation process and remain there during the drying step (Kadow et al., 2013; Ho et al., 2014, 2018; Sukha et al., 2014; Chetschik et al., 2018; Castro-Alayo et al., 2019; Rottiers et al., 2019; De Vuyst and Leroy, 2020). Hence, distinction has to be made between endogenous and microbially produced metabolites in both pulp and beans, an underrepresented approach (De Vuyst and Leroy, 2020). Also, the drying step as part of the curing process has to be examined in more detail. Finally, starter culture studies with the Trinitario cocoa variety are scarce, given its basic fine flavor quality (Cevallos-Cevallos et al., 2018; Ho et al., 2018; Castro-Alayo et al., 2019; Rottiers et al., 2019).

To estimate the contribution and impact of candidate starter culture strains, hence supporting their rational selection, fine-scale monitoring of the microbial communities is necessary, ideally at strain level, as it has not always been clear if the success of a starter culture-initiated cocoa fermentation process could indeed be ascribed to the strains inoculated or to the members of the background microbiota. Up to now, a number of culture-dependent (e.g., selective plating and incubation followed by isolate identification) and/or -independent techniques [e.g., denaturing gradient gel electrophoresis (DGGE) of rRNA-targeted polymerase chain reaction (PCR) amplicons (Nielsen et al., 2005; Camu et al., 2007), quantitative PCR (qPCR; Batista et al., 2015; Visintin et al., 2017), and pulsed-field gel electrophoresis (PFGE; Jespersen et al., 2005; Crafack et al., 2013)] have been used, albeit that these techniques can only discriminate microbial communities at species level at best. More recent techniques, such as amplicon and shotgun metagenomic sequencing, have been shown to allow a deeper and more accurate analysis of the microbiota of cocoa fermentation ecosystems (Garcia-Armisen et al., 2010; Illeghems et al., 2012; Mota-Gutierrez et al., 2018; Serra et al., 2019; Lima et al., 2020; Pacheco-Montealegre et al., 2020). Furthermore, the application of new approaches to assist the microbial ecosystem composition based on high-throughput amplicon sequencing and applying the concept of amplicon sequence variants (ASVs) gives a higher resolution than the operational taxonomic unit (OTU)-based approach used in the past, thereby increasing the information on species and even strain level that can be retrieved from a complex microbial environment, such as fermented foods (Callahan et al., 2017; Zhang et al., 2019). Indeed, ASVs are more accurate than OTUs because of the application of a quality-aware model of Illumina amplicon errors (Callahan et al., 2016). Similar microbial ecosystem members, clustered together by a dissimilarity threshold and assigned to a same taxon, may in fact belong to subpopulations of that taxon, which can be resolved using ASVs. Thus, an ASV approach can potentially resolve differences of as little as one nucleotide between amplicon reads and, therefore, be used to probe strain-level variation (Callahan et al., 2016).

The present study aimed at a multiphasic analysis of the effects of two candidate yeast starter culture strains, belonging to the species *S. cerevisiae* and *P. kudriavzevii*, as part of three functional starter culture mixtures, on the microbial community dynamics and metabolite compositions of pulp and beans during cocoa fermentation processes performed in vessels with a Trinitario variety in Costa Rica. This study further aimed at confirming the prevalence of the inoculated yeast, LAB, and AAB strains at DNA level by comparing rRNA-PCR-DGGE community profiling data, commonly used to follow up starter cultures, with data obtained through a high-throughput amplicon sequencing-based approach, in particular by refining the ASVs corresponding with species of the microbial genera involved and aligning them to the genomes of the inoculated strains. Finally, VOCs that are of sensory importance were quantified during both the fermentation and drying steps.

## Materials and Methods

### Starter Culture Strains

**FIGURE 1:**
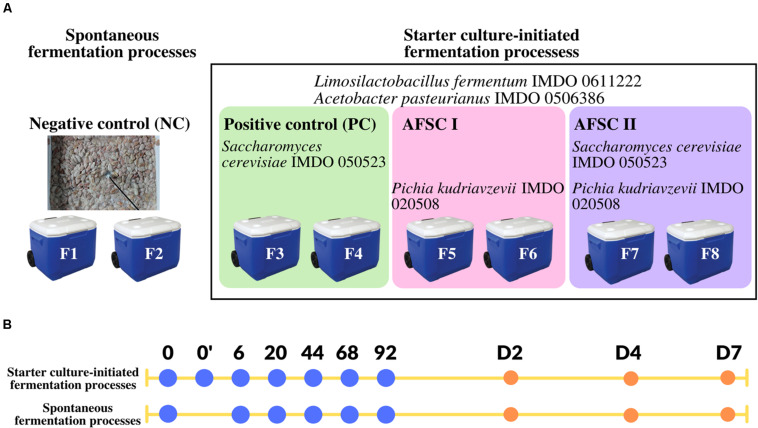
**(A)** Experimental set-up of duplicate 92 h cocoa fermentation processes, followed by 7 days of drying, carried out with 20 kg of cocoa pulp-bean mass (Trinitario cocoa from a plantation in Upala, Costa Rica) in plastic vessels at the campus of the Universidad Nacional de Costa Rica (Heredia, Costa Rica). The negative control NC represents the spontaneous cocoa fermentation processes (F1 and F2). The black frame represents the starter culture-initiated cocoa fermentation processes. Both bacterial strains, namely *Limosilactobacillus fermentum* IMDO 0611222 and *Acetobacter pasteurianus* IMDO 0506386, were inoculated in all starter culture-initiated cocoa fermentation processes. In addition, these starter culture-initiated cocoa fermentation processes were inoculated with either *Saccharomyces cerevisiae* IMDO 050523 [basic functional starter culture or positive control (PC), F3 and F4], *Pichia kudriavzevii* IMDO 050208 [adapted functional starter culture I (AFSC I), F5 and F6], or a combination of both yeast strains [adapted functional starter culture II (AFSC II), F7 and F8]. **(B)** The sampling time points of the cocoa fermentation processes performed are represented by blue dots (h of fermentation) and orange dots (days of sun-drying, D). The time point 0’ represents the sample taken right after inoculation of the starter cultures.

Microbial strains previously used as part of a functional starter culture [*S. cerevisiae* IMDO 050523, *Liml. fermentum* IMDO 0611222, and *A*. *pasteurianus* IMDO 0506386 (Lefeber et al., 2012)], and a yeast strain, namely *P. kudriavzevii* IMDO 020508 (isolate H2S5K8; Camu et al., 2007; Daniel et al., 2009), all previously isolated from a spontaneous cocoa fermentation process carried out in Ghana in 2004, were used throughout this study. The whole-genome sequences were available for *Liml. fermentum* IMDO 0611222 (Illeghems et al., 2015a), *A. pasteurianus* IMDO 0506386 (Illeghems et al., 2013), *S. cerevisiae* IMDO 050523 (16 chromosomes; C. Díaz-Muñoz, L. De Vuyst, and S. Weckx, unpublished results), and *P. kudriavzevii* IMDO 020508 (five chromosomes; C. Díaz-Muñoz, L. De Vuyst, and S. Weckx, unpublished results). The authenticity of all strains was checked by Sanger sequencing of the 16S rRNA gene for the bacterial strains and a region spanning the internal transcribed spacers 1 (ITS1) and 2 (ITS2) of the fungal ribosomal RNA transcribed unit for the yeast strains. These strains were used for the composition of three functional starter culture mixtures to be examined in the current study, namely a basic functional starter culture [further referred to as positive control (PC)], composed of *S. cerevisiae* IMDO 050523, *Liml. fermentum* IMDO 0611222 and *A. pasteurianus* IMDO 0506386, and two adapted functional starter cultures (AFSCs), further referred to as AFSC I (composed of *P. kudriavzevii* IMDO 020508, *Liml. fermentum* IMDO 0611222, and *A. pasteurianus* IMDO 0506386) and AFSC II (composed of *S. cerevisiae* IMDO 050523, *P. kudriavzevii* IMDO 020508, *Liml. fermentum* IMDO 0611222, and *A. pasteurianus* IMDO 0506386) (Figure 1).

All strains used throughout this study were stored at −80°C in glucose-yeast extract (GY) medium in the case of *S. cerevisiae* IMDO 050523 and *P. kudriavzevii* IMDO 020508, de Man-Rogosa-Sharpe (MRS) medium in the case of *Liml. fermentum* IMDO 0611222, and mannitol-yeast extract-peptone (MYP) medium in the case of *A. pasteurianus* IMDO 0506386, as reported previously (Moens et al., 2014).

### Biomass Production

For the production of biomass of each starter culture strain mentioned above, to be used as fermentation inocula, monoculture fermentations were carried out. Both yeast strains were grown in 1 L (*P. kudriavzevii* IMDO 020508) and 50 mL (*S. cerevisiae* IMDO 050523) of glucose-peptone-yeast extract (YPG) medium (De Roos et al., 2018). *Liml. fermentum* IMDO 0611222 was grown in 50 mL of modified MRS (mMRS) medium (Lefeber et al., 2012). *A. pasteurianus* IMDO 0506386 was grown in 50 mL of modified yeast extract-glucose-mannitol (mYGM) medium (Lefeber et al., 2012). For inoculum build-up, the strains were transferred three times in the respective media and grown at 30°C for 24 h (*S. cerevisiae* IMDO 050523, *P. kudriavzevii* IMDO 020508, and *A. pasteurianus* IMDO 0506386) or 16 h (*Liml. fermentum* IMDO 0611222). The volume transferred was always 1% (v/v) for all strains, except for *A. pasteurianus* IMDO 0506386 [5% (v/v)].

### Vessel Fermentation Processes

Eight cocoa fermentation processes were performed in 28-L plastic vessels that possessed an opening at the bottom to release the sweatings, each containing 20 kg of cocoa pulp-bean mass from fresh-harvested cocoa pods from a plantation of a Trinitario variety (Upala, Costa Rica). These vessel fermentation processes consisted of duplicates of a spontaneous fermentation process that served as a negative control (NC; F1 and F2) and three fermentation processes with the functional starter culture mixtures mentioned above, referred to as F3 and F4 (PC), F5 and F6 (AFSC I), and F7 and F8 (AFSC II) (Figure 1A). The biomass of the strains obtained as mentioned above was inoculated such that initial cell densities of 4.0 and 5.0 log [colony-forming units (CFU)/g] in the well-mixed cocoa pulp-bean mass were obtained for yeasts and both LAB and AAB, respectively.

All cocoa fermentation processes were performed for 92 h, based on the optimum fermentation duration for the basic functional starter culture PC (Lefeber et al., 2012). After the fermentation step, the cocoa beans were sun-dried on a woven polypropylene shadecloth on a cement patio for 7 days. The fermentation and drying steps were performed on the campus of the Universidad Nacional de Costa Rica (Heredia, Costa Rica) in November 2017 (transition between wet and dry season). Samples were taken after 0 (before inoculation), 0’ (after inoculation), 6, 20, 44, 68, and 92 h of fermentation and on day 2, 4, and 7 of the drying step (Figure 1B). After sampling, the cocoa pulp-bean mass in the vessels and the drying cocoa beans on the shadecloth were mixed. One part of the samples was used for immediate plating for microbial enumeration (only the samples from the fermentation step); another part was immediately frozen at −20°C for subsequent shipping on dry ice to Belgium for further culture-independent and metabolite target analyses.

### Online Monitoring of Temperature and pH

The temperature and pH of the cocoa pulp-bean mass were monitored on-line during the fermentation step in one of the duplicates of the different cocoa fermentation processes carried out. To this end, a WTW pH 3110 data logger (Xylem Analytics, Weilheim, Germany) was used. The measurements were stored every 30 min.

### Off-Line Monitoring of the Microbial Community Dynamics and Species Diversity

#### Selective Plating and Incubation for Colony Enumeration

To estimate the microbial cell densities at each time point of the sampling during the fermentation step of the eight cocoa fermentation processes examined, a culture-dependent analysis was performed, as described previously (Camu et al., 2007), with minor modifications. Approximately 20 g of cocoa pulp-bean mass were brought into a stomacher bag, diluted with 180 mL of saline (NaCl, 8.5 g/L; Merck, Darmstadt, Germany), and mixed manually. Tenfold serial dilutions were made for selective plating on YPG agar medium supplemented with chloramphenicol (200 mg/L; Merck) to determine the presumptive yeast counts, MRS agar medium supplemented with amphotericin (5 mg/L; Merck) and cycloheximide (200 mg/L; Merck) to determine the presumptive LAB counts, and mDMS agar medium supplemented with amphotericin (5 mg/L) and cycloheximide (200 mg/L) to determine the presumptive AAB counts. After incubation at 30°C for 48, 72, and 96 h, respectively, the colonies on agar media harboring 30–300 of them were counted and expressed as log (CFU/g) of cocoa pulp-bean mass for each time point sampled. Subsequently, plate washes were prepared by the addition of 10 mL of saline to the YPG, MRS, and mDMS agar media of these countable plates. These agar plate washes were centrifuged at 4,600 × *g* for 20 min at 4°C to obtain cell pellets, which were stored at −20°C for further analysis.

#### rRNA-PCR-DGGE Community Profiling of Agar Plate Washes for Starter Culture Monitoring

Total genomic DNA of colonies from 162 plate washes of YPG, MRS, and mDMS agar media, corresponding with the 54 sampling points of the eight cocoa fermentation processes examined, was isolated and subjected to DGGE of PCR amplicons that were obtained by targeting the rRNA genes of the genomic DNA (rRNA-PCR-DGGE community profiling), in particular to monitor starter culture addition performance, as described previously (Comasio et al., 2019). Three pairs of primers, namely LAC1–LAC2 for LAB (targeting the V3–V4 region of the 16S rRNA gene; Walter et al., 2001), WBAC1–WBAC2 for both LAB and AAB (targeting the V7-V8 region of the 16S rRNA gene; Lopez et al., 2003) and NL1-LS2 for yeasts (targeting the D1 region of the 26S rRNA gene; Nielsen et al., 2005), were used for DNA amplification. To avoid early separation of double-stranded DNA during DGGE, a GC-rich sequence was attached to the LAC2, WBAC2, and LS2 reverse primers. The PCR programs used for the LAC/WBAC and NL1-LS2 primer pairs were as described previously (Nielsen et al., 2005; Camu et al., 2007). For bacterial 16S rRNA-PCR-DGGE community profiling, the PCR amplicons were separated in an acrylamide gel containing gradients of 35–60% (LAC primer pair) and 45–70% (WBAC primer pair) of formamide as denaturing agent (Garcia-Armisen et al., 2010). For eukaryotic 26S rRNA-PCR-DGGE community profiling, a gradient of 35–60% of formamide was used (Papalexandratou and De Vuyst, 2011). To facilitate gel band identification, a reference ladder containing PCR amplicons obtained by using the primers mentioned above in a dedicated PCR with genomic DNA from pure cultures of the strains *A. pasteurianus* IMDO 0506386, *H. opuntiae* IMDO 040108, *Kluyveromyces marxianus* Y40, *Lacp. plantarum* IMDO 03P80, *Liml. fermentum* IMDO 0611222, *S. cerevisiae* IMDO 050523, and *P. kudriavzevii* IMDO 020508 was separated through DGGE simultaneously. To confirm the identity of the microorganisms corresponding with dedicated gel bands, these bands were excised, the DNA eluted and further amplified, and finally sequenced in a commercial facility (VIB genomics CORE, Antwerp, Belgium), as described previously (Camu et al., 2007). Percentages of identity with the closest known relatives of the partial sequences and accession numbers of hits from the GenBank database of the National Center for Biotechnology Information (NCBI, Bethesda, MD, United States) are reported.

#### Amplicon Sequencing for Microbial Species Diversity, Microbial Community Dynamics, and Starter Culture Monitoring

To estimate the microbial species diversity and community dynamics and to monitor the growth of the starter cultures added, a culture-independent analysis making use of whole-community DNA and applying an ASV approach was performed.

##### Total DNA extraction

Total genomic DNA of 54 cocoa pulp-bean mass samples covering all sampling points of the fermentation step of all cocoa fermentation processes performed was subjected to a metagenetic analysis. Therefore, cell pelleting and metagenomic DNA extraction was performed as described previously (De Bruyn et al., 2017). Minor modifications were applied to particularly remove contaminating plant material. Therefore, per sample, approximately 20 g of cocoa beans with surrounding pulp of the frozen samples were thawed and subsequently shaken (manually and vortexing) with 10 mL of saline. Beans were then removed manually, and the remaining pulp was centrifuged at 1,000 × *g* for 10 min at 4°C. The supernatant was separated from a thick pellet of mostly plant material by decantation, and subsequently filtered through a 20-μm pore-size Steriflip filter (Merck). The filtered supernatant was subjected to a second centrifugation step at 4,000 × *g* for 20 min at 4°C to have a consistent presumed cell pellet. This pellet was resuspended in at least 1 mL of saline and aliquoted over few 1.5-mL LoBind Eppendorf tubes (Eppendorf, Hamburg, Germany), whose number depended on the size of the pellet. Finally, the resuspended mixture was centrifuged at 6,000 × *g* for 10 min at 4°C, the supernatant was discarded, and the pellet was resuspended in 200 μL of glycerol-saline solution [glycerol (Merck), 50% (v/v); saline, 50% (v/v)]. These samples were frozen and stored at −80°C until further use.

Once thawed, the samples were used for the preparation of cell pellets by centrifugation at 6,000 × *g* for 10 min. Subsequently, an optimized cell lysis protocol was executed. First, yeast cell lysis was performed by resuspending the cell pellets in 600 μL of sorbitol buffer [21% (m/v) sorbitol (VWR International, Radnor, PA, United States) and 50 mM Tris base (Calbiochem, San Diego, CA, United States); pH 7.5], containing lyticase (0.2 U; Merck), Zymolyase (200 U; G-Biosciences, St. Louis, MO, United States), and β-mercaptoethanol (1.23 μL; Merck), and incubating the mixture at 30°C for 1 h, mixing it gently in-between. After this yeast cell lysis step, the mixtures were subjected to bacterial cell lysis after centrifugation at 6,000 × *g* for 10 min, resuspension of the pellets in 400 μL of STET buffer [8% (m/v) sucrose (Merck), 50 mM Tris base (Calbiochem), 50 mM ethylenediaminetetraacetic acid (EDTA; Merck), 5% (v/v) Triton X-100 (Merck); pH 8.0], containing mutanolysin (0.1 U; Merck) and lysozyme (8 mg; Merck), and incubation at 37°C for 1 h. Next, 40 μL of a 20% (m/v) sodium dodecyl sulfate solution (Merck) was added, together with 0.2 g of sterile acid-washed 200-μm glass beads (Merck), and the mixtures were vortexed intensively for 2 min to accomplish chemical and mechanical cell lysis. This was followed by proteolysis by the addition of 50 μL of a proteinase K solution [2 mg of proteinase K (Merck) in 1 mL of TE buffer [50 mM Tris base (Calbiochem) and 1 mM EDTA (Merck)] and incubation of the mixtures at 56°C for 1 h. Subsequently, 100 μL of a 5 M NaCl (Merck) solution was added, together with 80 μL of a 10% (m/m) cetyltrimethyl ammonium bromide (CTAB; Merck) solution, to facilitate the removal of contaminating polysaccharides. The mixtures were then vortexed and incubated at 65°C for 10 min. To extract the total DNA, 600 μL of a chloroform-phenol-isoamyl alcohol solution (49.5:49.5:1.0; Merck) was added, and the lysates were shaken vigorously for 5 min. Then, these solutions were centrifuged at 18,000 × *g* for 5 min in 2-mL vials (Phase Lock Gel Heavy; 5 prime, Hilden, Germany). Afterward, the resulting aqueous phase solutions were subjected to a RNAse treatment, for which 10 μL of a RNAse solution (10 mg/mL; Thermo Fisher Scientific, Waltham, MA, United States) was added and the mixtures were incubated at 37°C for 10 min. Finally, these mixtures were purified using the DNeasy blood and tissue kit (Qiagen, Hilden, Germany) according to the manufacturer’s instructions. The DNA concentrations were measured using fluorimetry (Qubit, Thermo Fisher Scientific).

##### Amplicon sequence analysis

Amplicon generation Amplification of the V4 hypervariable region of the bacterial 16S rRNA gene was performed with primer pair F515-R806 (Caporaso et al., 2011) and that of the ITS1 region of the fungal ribosomal RNA transcribed unit with primer pair BITS1-B58S3 (Bokulich and Mills, 2013). This amplification and all subsequent steps were performed as described previously (De Bruyn et al., 2017). The amplicon sequences are available under the accession numbers ERX4611870–ERX4611923 in the European Nucleotide Archive of the European Bioinformatics Institute (ENA/EBI).

Microbial community and species diversity dynamics Amplicon sequences were quality-filtered and further trimmed (only for the V4 amplicons) to infer the ASVs by using the DADA2 package (version 1.10.1; Callahan et al., 2017). For the V4 amplicons, the following filtering parameters were applied: maxN = 1, truncQ = 2, maxEE = (2,3), minLen = 50, and truncLen = 250. Then, the filtered reads were put into the DADA2’s parametric error model. For the ITS1 region amplicons, the parameters maxN = 1, truncQ = 2, maxEE = 5, and minLen = 50 were applied. The filtered ITS1 reads were not trimmed because of the expected length variability for yeasts and filamentous fungi. Only forward reads were used to generate the fungal ASVs, as the increased and variable length of the ITS1 region sequenced did not allow an accurate merging of the forward and reverse reads. Taxonomy was assigned with the SILVA database (version 132; Quast et al., 2013) for the bacterial ASVs and the UNITE database (version 02.02.2019; Kõljalg et al., 2013) for the fungal ASVs. Only genera with relative abundances above 0.5% in at least one of the 54 samples are reported.

Monitoring of the starter cultures applied For a more detailed analysis of the ASVs, the basic local alignment search tool (BLAST, version 2.2.30; Altschul et al., 1997) was used to assign the identities of the closest known relatives (type strains) from the GenBank database of NCBI. Identifications were only reported if the identities were above 97%. In addition, the same tool was used to align the ASVs against the genome sequences of the inoculated strains (*Liml. fermentum* IMDO 0611222, *A. pasteurianus* IMDO 0506386, *S. cerevisiae* IMDO 050523, and *P. kudriavzevii* IMDO 020508). As there were several ITS1 regions spread over three chromosomes in the case of the genome of *P. kudriavzevii* IMDO 020508, the best hits for those ASVs for each chromosome were reported.

### Off-Line Monitoring of the Substrate Consumption and Metabolite Production Dynamics

To determine the concentrations of the substrates and metabolites of both the cocoa pulp and cocoa beans as a function of time, a metabolite target analysis approach was performed. Therefore, the cocoa beans were separated from the pulp (and testa) of each thawed sample. The pulp and beans obtained were separately frozen in liquid nitrogen (Air Liquide, Louvain-la-Neuve, Belgium) and grinded into fine powders with a coffee grinder (DeLongi KG49, Treviso, Italy). Extracts of these cocoa pulp and cocoa bean powders were prepared as follows (Zhang et al., 2019). Aqueous extracts for quantification of substrates and metabolites (except for VOCs) were made at room temperature by mixing 0.2 g of these powders and 14 mg of EDTA (Merck) in 5 mL of an ascorbic acid solution (2.0 mg/mL; VWR International). These mixtures were then vortexed, shaken at 40 rpm for 25 min, and centrifuged at 4,700 × *g* for 10 min. The supernatants were transferred to 2-mL Eppendorf tubes and stored at −20°C until further analysis. Ethyl acetate extracts for quantification of VOCs were used after an optimization process of the extraction solvent, thereby comparing ethyl acetate (Merck), acetone (VWR International), and methanol (Merck) to select the solvent that allowed the detection of VOCs with the highest reproducibility based on relative standard deviations. Ethyl acetate extracts were made freshly at room temperature in triplicate by mixing 2 g of the powders mentioned above with 10 mL of ethyl acetate (Merck). These mixtures were then vortexed, shaken at 40 rpm for 20 min, and centrifuged at 4,000 × *g* for 20 min. The supernatants were filtered with a Millex Syringe Driven Filter Unit (Merck) and immediately used for further analysis.

#### Simple Carbohydrates and Sugar Alcohols

The concentrations of simple carbohydrates (i.e., fructose, glucose, and sucrose) and sugar alcohols (i.e., glycerol and mannitol) were quantified in the aqueous extracts in triplicate by high-performance anion exchange chromatography with pulsed amperometric detection (HPAEC-PAD), using ICS 3000 chromatograph systems equipped with a CarboPac PA-20 and CarboPac MA-1 column, respectively (Thermo Fisher Scientific), as described previously (De Bruyn et al., 2017). Quantification was performed by external calibration, including an internal standard [IS; solution of 0.02 g of rhamnose (Merck) per liter of acetonitrile (Thermo Fisher Scientific)].

#### Ethanol, Acetic Acid, and Acetoin

The concentrations of ethanol, acetic acid, and acetoin were quantified in the aqueous extracts in triplicate by gas chromatography with flame ionization detection (GC-FID), using a Focus GC (Interscience, Breda, Netherlands) equipped with a Stabilwax-DA column (Restek, Bellefonte, PA, United States) and a FID-80 detector (Interscience), as described previously (De Bruyn et al., 2017). Quantification was performed by external calibration, including 1-butanol as IS [solution of 638 mL of acetonitrile (Thermo Fisher Scientific), 250 mL of ultrapure water (MilliQ; Merck), 12 mL of formic acid (Merck), and 250 μL of 1-butanol (Merck)].

#### Organic Acids

The concentrations of organic acids (i.e., citric acid, gluconic acid, lactic acid, malic acid, and succinic acid) were quantified in the aqueous extracts in triplicate by ultra-performance liquid chromatography with tandem mass spectrometry (UPLC-MS/MS), using an Acquity UPLC system equipped with a HSS T3 column coupled to a TQ tandem mass spectrometer (Waters, Milford, MA, United States), as described previously, except for the mobile phase used (De Bruyn et al., 2017). The latter consisted of an ultrapure water (MilliQ)-methanol (Merck) mixture (980:20, v/v) with 0.2% (v/v) formic acid (Merck) (eluent A) and an ultrapure water-methanol mixture (50:950, v/v) with 0.2% (v/v) formic acid (eluent B). Quantification was performed by external calibration.

#### Volatile Organic Compounds

##### Screening

To make a selection of VOCs to be quantified in both the cocoa pulp and cocoa bean samples, first headspace/solid-phase microextraction coupled to gas chromatography with time-of-flight mass spectrometry (HS/SPME-GC-TOF-MS) was used as a screening tool. Cocoa pulp and cocoa bean powders (0.5 g), supplemented with 10 μL of a solution of 40 ppm of Toluene-D8 (Merck) added as IS, were incubated in 10-mL screw-cap headspace vials at 40°C for 10 min and subsequently brought into contact with a SPME fiber [divinylbenzene/carboxen/polydimethylsiloxane (DVB/CAR/PDMS), 50/30 μm; Supelco, Merck] with agitation at 250 rpm for 45 min. This was done in triplicate; a compound that was found at least two times was considered as being present. The VOCs were thermally desorbed from the SPME fiber at 250°C and resolved with a fused silica capillary Stabilwax-MS column (Restek) placed in a Trace 1300 gas chromatograph (Thermo Fisher Scientific). The split/splitless injector was set at a split flow of 50 mL/min. Helium (Praxair, Danbury, CT, United States) was used as carrier gas at a flow rate of 1 mL/min. The oven temperature was programmed initially at 40°C for 1.5 min, then raised to 225°C at 10°C/min, and finally held at this temperature for 15 min. The transfer line and ion source temperatures of the Bench TOF-HD mass spectrometer (Markes International, Llantrisant, Wales, United Kingdom), which was operated in the electron impact mode with an electron energy of 70 eV, were set at 250 and 220°C, respectively. Scanning was performed at a *m/z* range from 35 to 400 with a solvent delay of 2 min. The NIST14 library (National Institute of Standards and Technology, Gaithersburg, MD, United States) was used for peak identification, considering a match factor above 750. Also, the Kováts retention index was used (Afeefy et al., 2017). The PubChem database (NCBI; Kim et al., 2019) was consulted to allocate flavor notes to the VOCs identified.

##### Quantification

Twenty-nine VOCs determined through HS/SPME-GC-TOF-MS were then chosen for quantification, based on their frequent occurrence in cocoa fermentation processes and their presumed beneficial contributions to chocolate flavor (Frauendorfer and Schieberle, 2008; Aculey et al., 2010; Owusu et al., 2012; Rodriguez-Campos et al., 2012; Crafack et al., 2013; De Vuyst and Leroy, 2020). The concentrations of these VOCs were quantified in the ethyl acetate extracts (see section “Off-Line Monitoring of the Substrate Consumption and Metabolite Production Dynamics”) by liquid injection gas chromatography with tandem mass spectrometry (LI-GC-MS/MS). A Trace 1300 gas chromatograph equipped with a DBwax-etr column (Thermo Fisher Scientific) and a TriPlus RSH autosampler (Thermo Fisher Scientific) coupled to a TSQ 8000 EVO triple quadrupole mass spectrometer (Interscience) was used. The extracts were mixed with an IS solution of Toluene-D8 (0.5%, v/v; Merck). Finally, 400 μL of this solution was transferred to glass vials (Macherey-Nagel, Düren, Germany) for injection of 1 μL into the column, applying a split flow of 5 mL/min. Helium (Praxair) with a constant flow rate of 1 mL/min was used as carrier gas. Argon (Praxair) was chosen as collision gas. The GC oven temperature was programmed initially at 40°C for 1.5 min, then raised to 225°C at 10°C/min, and finally held at this temperature for 15 min. The total run time was 35 min. The inlet temperature was set at 250°C. The mass spectrometer operated in the electron impact mode with an electron energy of 70 eV. The transfer line and ion source temperatures were set at 250 and 280°C, respectively. Quantification was performed with external calibration.

### Statistical Analysis

For the statistical processing of all kinds of data obtained, the software RStudio (version 3.4.4; RStudio Team, 2018) was used. For each independent analysis aiming at comparing two groups of data, a Shapiro–Wilk test was applied to test the normality of the samples and an *F* test was applied to compare the variance of the two sets of data. Based on the results of these tests, a regular *t*-test, Welch-corrected *t*-test, or Wilcoxon rank-sum test was performed to determine significant differences (stats package, version 3.6, R Core Team, 2018).

The alpha- and beta-diversities of the amplicon sequence data were examined (Vegan package, version 2.5-6; Oksanen et al., 2019). To determine whether or not the application of the starter culture mixtures effectively influenced the microbial communities involved in the cocoa fermentation processes performed, the alpha-diversity of the ASVs belonging to species of the inoculated genera was considered. Thus, the relative abundances of each ASV at every time point were summed and analyzed as a unique entity to have a representation of the diversity (Shannon index) and evenness (Pielou index) of each cocoa fermentation process taken as a whole. To assess the beta-diversity, a pairwise PERMANOVA was performed to determine differences in bacterial and yeast compositions among the spontaneous and starter culture-initiated cocoa fermentation processes performed (both ASVs and counts).

Two Spearman correlation analyses (*p* < 0.05) were performed, one between the main microbial genera present in the pulp (in the case of >5% relative abundance of ASVs in at least one sample), their substrates consumed and their metabolites produced, and another between these genera and the VOCs quantified in the cocoa pulp, using the Hmisc package (version 4.2-0; Harrell and Dupont, 2019) to calculate the correlation and corrplot (0.84; Wei and Simko, 2017) for the visualization of the matrix. A hierarchical clustering method with single agglomeration was applied.

To assess a comparison of the VOC compositions of the cocoa pulp and beans during the fermentation step and of the beans during the drying step as a function of time, as well as an influence of the functional starter culture mixtures used on the VOCs quantified, two principal component analyses (PCAs) were performed and plotted using ggplot2 (Wickham, 2016). Each PCA was based on a covariance matrix of the concentrations of 29 VOCs quantified in the cocoa pulp and bean samples of all cocoa bean curing processes performed. The choice of the number of the principal components (PCs) included was based on a Scree plot. The quantitative data were normalized by means of *Z*-score transformations to generate two heatmaps using the ComplexHeatmap package (version 2.0.0; Gu et al., 2016). Hierarchical clustering analysis was based on the Ward’s method (clustering method set to “Ward.D2”). The Euclidean distance calculation method was applied to assess the similarity between samples.

## Results

### Course of pH and Temperature

**FIGURE 2:**
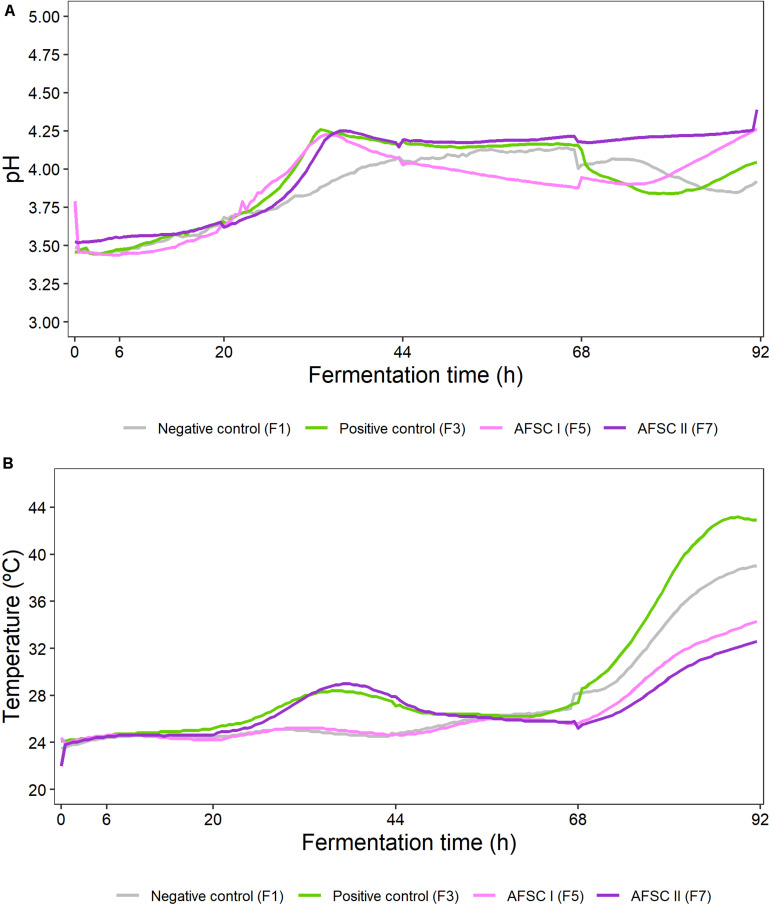
Course of pH **(A)** and temperature **(B)** during 92 h Costa Rican cocoa fermentation processes, whose set-up is explained in the legend of Figure 1. Only one of the biological replicates of each fermentation process was monitored online (number of vessel used is indicated between brackets). The *X*-axis ticks represent sampling time points when the fermenting cocoa pulp-bean mass was mixed, except after 6 h of fermentation.

The initial pH and temperature of the cocoa pulp-bean mass of the four Trinitario cocoa fermentation processes examined were 3.5 and 23.5°C (NC), 3.5 and 24.1°C (PC), 3.8 and 24.4°C (AFSC I), and 3.5 and 22.0°C (AFSC II) (Figure 2).

The pH of all cocoa fermentation processes increased from the start till 33–36 h of fermentation for the starter culture-initiated ones (PC, AFSC I, and AFSC II). Such increase was not equally pronounced for the spontaneous one (NC). Whereas the pH of the spontaneous cocoa fermentation process NC slightly increased further till 66 h and then decreased, that of the PC and AFSC II fermentation processes stabilized till 66 h or the end, respectively. In contrast, the pH of the AFSC I fermentation process decreased from 33 to 68 h and then increased again till the end. That of the PC fermentation process further showed a decrease (after 66 h) followed by an increase (from 80 h till the end). This resulted in final pH values of 3.9 (NC), 4.1 (PC), 4.3 (AFSC I), and 4.4 (AFSC II).

An increase of the temperature along the cocoa fermentation processes occurred in all cases, which was most pronounced after approximately 68 h of fermentation, resulting in final values of 42.9°C (PC), 39.0°C (NC), 34.3°C (AFSC I), and 32.6°C (AFSC II). The initial increase was slow and continuous in the case of the NC and AFSC I fermentation processes. The temperature profile showed an intermediate maximum after 37 h of fermentation in the case of the PC and AFSC II fermentation processes, which were inoculated with *S. cerevisiae* IMDO 050523.

The only significant difference (*p* < 0.05) in the pH profiles of the cocoa fermentation processes examined was found for the AFSC II fermentation process compared with the NC (*p* = 0.0003), PC (*p* = 0.0052), and AFSC I (*p* = 0.0003) fermentation processes. Regarding the temperature profiles, all were significantly different from each other (*p* < 0.05), except for the AFSC II fermentation process compared to the NC (*p* = 0.1513) and AFSC I (*p* = 0.1043) fermentation processes.

### Culture-Dependent Microbial Community Dynamics

**FIGURE 3:**
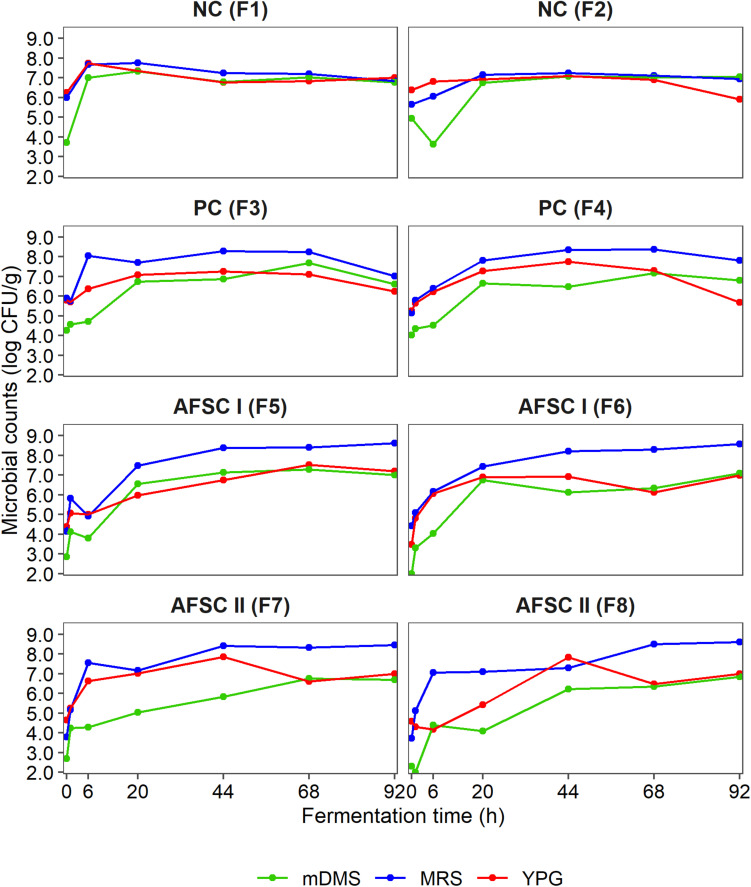
Dynamics of the presumptive yeasts, lactic acid bacteria (LAB), and acetic acid bacteria (AAB), expressed as counts on yeast-peptone-glucose (YPG) agar medium, de Man-Rogosa-Sharpe (MRS) agar medium, and modified deoxycholate-mannitol-sorbitol (mDMS) agar medium, respectively, during 92 h Costa Rican cocoa fermentation processes carried out in eight different vessels. The type of fermentation process (F1–F8) and sampling are as explained in the legend of Figure 1.

The initial counts of the presumptive yeasts, LAB, and AAB in the cocoa pulp-bean mass were 6.3 log (CFU/g), 5.9 log (CFU/g), and 4.7 log (CFU/g), respectively, for the NC; 5.6 log (CFU/g), 5.7 log (CFU/g), and 4.2 log (CFU/g) for the PC; 4.1 log (CFU/g), 4.3 log (CFU/g), and 2.6 log (CFU/g) for the AFSC I; and 4.6 log (CFU/g), 3.7 log (CFU/g), and 2.5 log (CFU/g) for the AFSC II cocoa fermentation processes (Figure 3). The starter culture mixtures were inoculated at initial cell densities of 4.3 log (CFU/g) of cocoa pulp-bean mass for *S. cerevisiae* IMDO 050523, 4.4 log CFU/g for *P. kudriavzevii* IMDO 020508, 5.0 log CFU/g for *A. pasteurianus* IMDO 0506386, and 5.3 log CFU/g for *Liml. fermentum* IMDO 0611222. Upon fermentation, the presumptive LAB always represented the microbial group with the highest counts, reaching maximum average values of 7.7 log (CFU/g) for the spontaneous cocoa fermentation processes (NC) and 8.4 log (CFU/g) (PC), 8.6 log (CFU/g) (AFSC I), and 8.6 log (CFU/g) (AFSC II) for the starter culture-initiated ones. However, no significant differences occurred in the presumptive LAB populations between spontaneous and starter culture-initiated cocoa fermentation processes (*p* > 0.05). Further, the course of the counts of the presumptive yeasts, representing the second most abundant microbial group throughout the fermentation processes, was comparable (*p* > 0.05) for the spontaneous and starter culture-initiated ones, albeit that a slower trend was seen for the AFSC ones, with yeast counts of 6.5 log (CFU/g) (NC), 6.0 log (CFU/g) (PC), 7.1 log (CFU/g) (AFSC I), and 7.0 log (CFU/g) (AFSC II) after 92 h of fermentation. The presumptive AAB populations reached counts higher than 6.0 log (CFU/g) after 20 h of fermentation, except for AFSC II, for which these counts were reached toward the end of the fermentation processes. The highest AAB counts were reached after 68 h of fermentation in the NC and PC fermentation processes [7.0 log (CFU/g) and 7.4 log (CFU/g), respectively] and after 92 h of fermentation in the AFSC I and AFSC II fermentation processes [7.1 log (CFU/g) and 6.8 log (CFU/g), respectively]. A significant difference in the AAB populations (*p* < 0.05) could be found between the spontaneous and starter culture-initiated fermentation processes, although the PERMANOVA did not indicate any difference (*p* > 0.05) among the four different cocoa fermentation processes carried out.

### rRNA-PCR-DGGE Community Profiling for Starter Culture Monitoring

Semi-quantitative rRNA-PCR-DGGE community profiling based on total genomic DNA from agar plate washes of the presumptive yeast, LAB, and AAB populations showed the main microbial taxa present in the cocoa fermentation processes performed (Supplementary Figure 1). Concerning yeasts (Supplementary Figure 1A), the presence of *Hanseniaspora* was demonstrated. Toward the end of the spontaneous fermentation processes (NC), a *S. cerevisiae*-derived PCR amplicon appeared, replacing the *Hanseniaspora* ones as a function of time. This shift from *Hanseniaspora* to *S. cerevisiae* occurred earlier in the PC fermentation processes, which were inoculated with *S. cerevisiae* IMDO 050523. PCR amplicons corresponding to *P. kudriavzevii* were present in both AFSC fermentation processes, which was in line with the inoculation of *P. kudriavzevii* IMDO 020508. PCR amplicons corresponding to *S. cerevisiae* were also found in the AFSC II cocoa fermentation processes that were started with *S. cerevisiae* IMDO 050523 too.

Concerning LAB (Supplementary Figure 1B), PCR amplicons corresponding to *Liml. fermentum* were found in all starter culture-initiated cocoa fermentation processes, which was in line with the inoculation of *Liml. fermentum* IMDO 0611222. Regarding the NC fermentation processes, PCR amplicons corresponding to *Weissella fabalis*/*beninensis*/*ghanensis* were found throughout fermentation, representing this LAB species as the main one. This PCR amplicon was also found in the beginning of the starter culture-initiated fermentation processes but was always overthrown by *Liml. fermentum* as fermentation proceeded. In the NC fermentation processes, this *Weissella* species was replaced by *Lactiplantibacillus pentosus/plantarum/paraplantarum* at the end.

With regard to the AAB communities (Supplementary Figure 1C), the presence of PCR amplicons showed that the AAB communities mainly consisted of *A. pasteurianus* in all starter culture-initiated cocoa fermentation processes and only at the end during the spontaneous cocoa fermentation processes NC.

### Amplicon Sequence Variant Approach for Microbial Community Dynamics, Microbial Species Diversity, and Starter Culture Monitoring

#### Microbial Community and Species Diversity Dynamics

**FIGURE 4:**
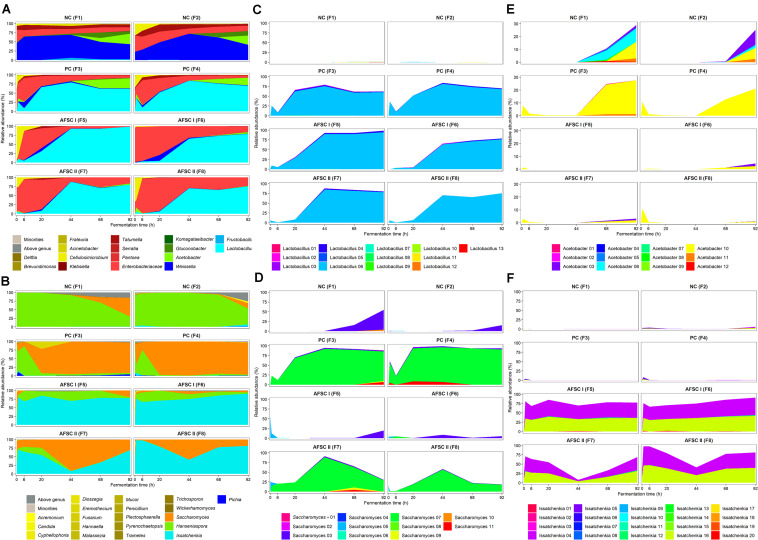
Microbial community and species dynamics, expressed as relative abundances based on the amplicon sequence variants (ASVs) of the V4 region of the 16S rRNA gene for bacteria **(A)** and the internal transcribed spacer (ITS) region ITS1 of the rRNA transcribed unit for yeasts **(B)** during 92 h Costa Rican cocoa fermentation processes carried out in eight different vessels. The type of fermentation process (F1–F8) and sampling are as explained in the legend of Figure 1. Microbial groups with relative abundances below 0.5% are represented in the category ‘Minorities.’ ASVs that could not be allocated to genus level are represented in the category ‘Above genus.’ The *Enterobacteriaceae* family is indicated separately. Starter culture growth monitoring allocated the ASVs to specific strains of the genera *Lactobacillus* **(C)**, *Saccharomyces* **(D)**, *Acetobacter* **(E)**, and *Issatchenkia* **(F)**.

The ASV analysis performed on the V4 and ITS1 amplicon sequences obtained for all cocoa pulp-bean mass fermentation samples showed a complete picture of the microbial community dynamics throughout the cocoa fermentation processes performed (Figure 4). With regard to the bacterial communities, the beginning of all cocoa fermentation processes (first 24 h of fermentation) was characterized by the presence of enterobacteria (relative abundances of 6.2–96.5%), which were more abundant (*p* < 0.05) in all starter culture-initiated fermentation processes compared to the spontaneous ones (Figure 4A). However, no distinction could be made at genus level for certain ASVs (100% identity to *Tatumella*, *Pantoea*, *Klebsiella*, and *Erwinia*). After 20 h of fermentation, an increase of the relative abundances of different genera of LAB (relative abundances of 41.8–98.6%) occurred, becoming the most abundant microbial group in all fermentation processes. In accordance with the 16S rRNA-PCR-DGGE data, *Weissella* was the main LAB genus in the NC fermentation processes, reaching maximum relative abundances of 66.4% (F1) and 72.2% (F2) after 48 h of fermentation. The LAB fraction of all starter culture-initiated fermentation processes was characterized by high relative abundances of *Lactobacillus* from the beginning to the end of these fermentation processes, reaching maximum relative abundances after 44 h of fermentation in the case of F3 (79.6%), F4 (84.1%), and F7 (88.0%) or 92 h of fermentation in the case of F5 (98.6%), F6 (78.7%), and F8 (75.5%). Regarding the AAB communities, high relative abundances were only found late in the fermentation processes, being much more abundant in the NC (35.3 and 37.8% in F1 and F2, respectively) and PC fermentation processes (29.2 and 21.4% in F3 and F4, respectively) than in the AFSCs after 92 h of fermentation. However, the ASVs belonging to AAB in the NC fermentation processes were a consortium of *Acetobacter* (28.9 and 25.3% in F1 and F2, respectively) and *Gluconobacter* (6.5 and 12.5% in F1 and F2, respectively), whereas in the PC fermentation processes *Acetobacter* (27.6 and 21.1% in F3 and F4, respectively) was much higher in relative abundances than *Gluconobacter* (1.6 and 0.3% in F3 and F4, respectively).

Concerning the yeast communities, ASVs from the *Hanseniaspora* genus could be retrieved from all cocoa fermentation processes, in line with the 26S rRNA-PCR-DGGE community profiling (Figure 4B). These *Hanseniaspora* ASVs remained at high relative abundances in the NC fermentation processes until the end, albeit with a decreasing trend (from 99.9 to 29.8% in F1 and from 90.3 to 49.0% in F2). Toward the end, also *Saccharomyces* ASVs appeared at high relative levels (reaching 56.4 and 15.5% in F1 and F2, respectively, after 92 h of fermentation). The PC fermentation processes were completely represented by *Saccharomyces*, as shown by the high relative abundances of the ASVs belonging to this genus after 6 h of fermentation until the end (89.6 and 95.0% in F3 and F4, respectively, after 92 h of fermentation). ASVs belonging to the *Issatchenkia* genus were found in both AFSC fermentation processes (relative abundances ranging between 6.9 and 99.9% throughout the fermentation processes). A deeper analysis of those ASVs allowed their identification as *Issatchenkia orientalis*, which is a later synonym of *P. kudriavzevii* (Douglass et al., 2018). In the AFSC II fermentation processes, the most abundant genera were *Saccharomyces* (maximum relative abundances of 92.2 and 56.6% in F7 and F8, respectively, after 44 h of fermentation) and *Issatchenkia* (*P. kudriavzevii*; maximum relative abundances of 99.3 and 98.0% in F7 and F8, respectively, after 6 h of fermentation).

PERMANOVA to assess the beta-diversity showed that the microbial compositions of the spontaneous and starter culture-initiated cocoa fermentation processes were significantly different (*p* < 0.05) at the genus level. Moreover, the bacterial compositions of the three starter-culture initiated fermentation processes (PC, AFSC I, and AFSC II) were not significantly different (*p* > 0.05), indicating the efficacy of the addition of the bacterial starter culture strains. Regarding the yeast communities, all cocoa fermentation processes were significantly different from each other (*p* < 0.05), except for AFSC I *versus* AFSC II (*p* = 0.066), indicating an effect of the yeast composition of the starter culture mixtures added.

#### Monitoring of the Starter Culture Strains Added

The ASVs belonging to the genera corresponding with the species of the strains used as part of the starter culture mixtures were analyzed in more detail (Figures 4C–F). For each bacterial genus examined, there was one unique ASV at high relative abundance during all starter culture-initiated cocoa fermentation processes performed, which in most cases was different from the ASVs found in the NC fermentation processes, indicating the prevalence of the starter culture strain added. Of the 13 *Lactobacillus* and 12 *Acetobacter* ASVs detected, the *Lactobacillus* 06 and *Acetobacter* 10 ASVs were those found at the highest relative abundances in the fermentation processes inoculated with *Liml. fermentum* IMDO 0611222 and *A. pasteurianus* IMDO 0506386 (i.e., PC, AFSC I, and AFSC II), respectively (Figures 4C,E). A local alignment of these ASVs against the whole-genome sequences of the bacterial strains inoculated showed that only the *Lactobacillus* 06 and *Acetobacter* 10 ASVs possessed 100% identity with the corresponding region of the 16S rRNA gene of *Liml. fermentum* IMDO 0611222 and *A. pasteurianus* IMDO 0506386, respectively (Tables 1, 2). These ASVs were also present in the NC fermentation processes, albeit to a low extent (Figures 4C,E). Examination of the 11 ASVs belonging to the *Saccharomyces* genus also showed differences between the ASVs found in the fermentation processes inoculated with *S. cerevisiae* IMDO 050523 (i.e., PC and AFSC II), being mainly *Saccharomyces* 07, and those found in the fermentation processes that were not inoculated with this *Saccharomyces* strain (NC and AFSC I), being mainly *Saccharomyces* 03 and *Saccharomyces* 04 (Figure 4D). The *Saccharomyces* 07 ASV was 100% identical with the ITS1 region of the genome sequence of *S. cerevisiae* IMDO 050523 (chromosome XII; Table 3). However, also the *Saccharomyces* 08 ASV showed 100% identity with the ITS1 region of the genome sequence of the inoculated strain (chromosome XII, Table 3), but this ASV was 2 bp longer than the ITS1 region of the inoculated strain, indicating a difference between this sequence and that of the inoculated strain. The 20 *Issatchenkia* ASVs were identified as *P. kudriavzevii*, and two of them, namely the *Issatchenkia* 04 and *Issatchenkia* 16 ASVs (Figure 4F), were found at the highest relative abundances in those fermentation processes inoculated with *P. kudriavzevii* IMDO 020508 (i.e., AFSC I and AFSC II). The latter strain harbored eight different ITS1 sequences that were spread over 27 different loci on chromosomes I and III (data not shown). The *Issatchenkia* 04 ASV showed 100% identity with an ITS1 sequence on chromosomes I and III of the inoculated *Pichia* strain (Table 4). The *Issatchenkia* 16 ASV was 100% identical with a different ITS1 sequence on chromosome I. Those two variants co-occurred at the same ratio of relative abundances (average of 1.208 ± 0.150) throughout both AFSC fermentation processes. Furthermore, the *Issatchenkia* 05 ASV was only present in the NC, PC, and AFSC I cocoa fermentation processes (in the latter one only before inoculation of the starter culture mixtures), and showed 100% identity with another ITS1 sequence on chromosome I. Finally, the *Issatchenkia* 15 ASV was only detected in the 68 h fermentation sample from vessel F2 of the NC fermentation process in very low numbers, and also showed 100% identity with still another ITS1 sequence on chromosome I.

**TABLE 1:** Overview of the identification of the *Lactobacillus*-related amplicon sequence variants (ASVs; first column).

**ASV**	**Identity based on alignment against the NCBI nt database**	**Sequence identity (%) (*Limosilactobacillus fermentum* IMDO 0611222)**	**Non-identical nucleotides (*Limosilactobacillus fermentum* IMDO 0611222)**
*Lactobacillus* 01	*Lactobacillus fermentum*	98	6
*Lactobacillus* 02	*L. fermentum*	98	4
*Lactobacillus* 03	Uncultured bacterium*	96	9
*Lactobacillus* 04	*L. fermentum*	99	2
*Lactobacillus* 05	*L. fermentum*	99	1
*Lactobacillus* 06	*L. fermentum*	100	0
*Lactobacillus* 07	*L. fermentum*	98	5
*Lactobacillus* 08	Lactobacillus paracasei*	97	8
*Lactobacillus* 09	*L. fermentum*	98	4
*Lactobacillus* 10	*Lactobacillus pentosus/plantarum/paraplantarum*	93	17
*Lactobacillus* 11	*Lactobacillus cacaonum*	91	24
*Lactobacillus* 12	*Lactobacillus hayakitensis*	91	24
*Lactobacillus* 13	*L. pentosus/plantarum/paraplantarum*	92	19

*The second column shows the identification (≥97% sequence identity) of the ASVs based on sequence alignment against type strain sequences present in the non-redundant nucleotide (nt) database of the National Centre for Biotechnology Information (NCBI). If the best match was below 97% sequence identity, the sequence was compared to all sequences in the nt database and the result (≥97% sequence identity) is indicated with an asterisk. The third and fourth columns indicate the percentage sequence identity and number of non-identical nucleotides when aligned against the genome sequence of the starter culture strain Limosilactobacillus fermentum IMDO 0611222.*

**TABLE 2:** Overview of the identification of the *Acetobacter* amplicon sequence variants (ASVs; first column).

**ASV**	**Identity based on alignment against the NCBI nt database**	**Identity (%) *(Acetobacter pasteurianus* IMDO 0506386)**	**Non-identical nucleotides *(Acetobacter pasteurianus* IMDO 0506386)**
*Acetobacter* 01	*A. pasteurianus*	99	3
*Acetobacter* 02	*A. lambici/fabarum/okinawensis*	97	6
*Acetobacter* 03	*A. fabarum*	99	3
*Acetobacter* 04	*A. fabarum*	98	4
*Acetobacter* 05	*A. persici*	98	4
*Acetobacter* 06	*A. lambici/fabarum/okinawensis*	99	1
*Acetobacter* 07	*A. syzygii*	99	1
*Acetobacter* 08	*A. thailandicus*	99	2
*Acetobacter* 09	*A. orientalis/cibinongensis*	99	1
*Acetobacter* 10	*A. pasteurianus*	100	0
*Acetobacter* 11	*A. pasteurianus*	99	2
*Acetobacter* 12	*A. suratthaniensis/peroxydans/papaye*	98	5

*The second column shows the identification (≥97% sequence identity) of the ASVs based on sequence alignment against type strain sequences present in the non-redundant nucleotide (nt) database of the National Centre for Biotechnology Information (NCBI). The third and fourth columns indicate the percentage sequence identity and number of non-identical nucleotides when aligned against the genome sequence of the starter culture strain Acetobacter pasteurianus IMDO 0506386.*

**TABLE 3:** Overview of the identification of the *Saccharomyces* amplicon sequence variants (ASVs; first column).

**ASV**	**Identity based on alignment against the NCBI nt database**	**ASV length (bp)**	**Alignment length (bp) (*Saccharomyces cerevisiae* IMDO 050523)**	**Sequence identity (%) (*Saccharomyces cerevisiae* IMDO 050523)**	**Non-identical nucleotides (*Saccharomyces cerevisiae* IMDO 050523)**
*Saccharomyces* 01	<97%	301	280	95	15
*Saccharomyces* 02	*S. cerevisiae*	282	280	99	2
*Saccharomyces* 03	*S. cerevisiae*	282	279	99	1
*Saccharomyces* 04	*S. cerevisiae*	285	280	98	5
*Saccharomyces* 05	*S. cerevisiae*	283	277	98	6
*Saccharomyces* 06	*S. cerevisiae*	282	275	98	7
*Saccharomyces* 07	*S. cerevisiae*	282	282	100	0
*Saccharomyces* 08	*S. cerevisiae*	284	282	100	0
*Saccharomyces* 09	*S. cerevisiae*	282	282	99	1
*Saccharomyces* 10	*S. cerevisiae*	282	282	99	3
*Saccharomyces* 11	*S. cerevisiae*	282	282	99	2

*The second column shows the identification (≥97% sequence identity) of the ASVs based on sequence alignment against type strain sequences present in the non-redundant nucleotide (nt) database of the National Centre for Biotechnology Information (NCBI). The length in base pairs (bp) of the ASVs and the alignment length with S. cerevisiae IMDO 050523 are reported in the third and fourth columns. The fifth and sixth columns indicate the percentage sequence identity and number of non-identical nucleotides when aligned against the genome sequence of the starter culture strain S. cerevisiae IMDO 050523.*

**TABLE 4:** Overview of the identification of *Issatchenkia* amplicon sequence variants (ASVs; first column).

**ASV**	**Identity based on alignment against the NCBI nt database**	**Chromosome (*Pichia kudriavzevii* IMDO 020508)**	**Position (*Pichia kudriavzevii* IMDO 020508)**	**ASV length (bp)**	**Aligned length (bp) (*Pichia kudriavzevii* IMDO 020508)**	**Sequence identity (%) (*Pichia kudriavzevii* IMDO 020508)**
*Issatchenkia* 01	*P. kudriavzevii/I. orientalis*	I II III	5504 2748402 2588444	107	107 108 107	99 98 99
*Issatchenkia* 02	*P. kudriavzevii/I. orientalis*	I II III	5504 2748402 2588444	107	107 108 107	99 98 99
*Issatchenkia* 03	*P. kudriavzevii/I. orientalis*	I II III	5504 2748402 2588444	107	107 108 107	99 98 99
*Issatchenkia* 04	*P. kudriavzevii/I. orientalis*	I II III	5504 2748402 2588444	107	107 108 107	100 99 100
*Issatchenkia* 05	*P. kudriavzevii/I. orientalis*	I II III	3098052 2748402 2588444	106	106 108 107	100 98 99
*Issatchenkia* 06	*P. kudriavzevii/I. orientalis*	I II III	5504 2748402 2588444	107	107 108 107	99 98 99
*Issatchenkia* 07	*P. kudriavzevii/I. orientalis*	I II III	5504 2748402 2588444	107	107 108 107	99 98 99
*Issatchenkia* 08	*P. kudriavzevii/I. orientalis*	I II III	5504 2748402 2588444	107	107 108 107	99 98 99
*Issatchenkia* 09	P. kudriavzevii *	I II III	5504 2748402 2588444	107	107 108 107	97 96 97
*Issatchenkia* 10	*P. kudriavzevii/I. orientalis*	I II III	5504 2748402 2588444	107	107 108 107	99 98 99
*Issatchenkia* 11	P. kudriavzevii *	I II III	5504 2748402 2588444	107	107 108 107	97 96 97
*Issatchenkia* 12	P. kudriavzevii *	I II III	5504 2748402 2588444	107	107 108 107	96 95 96
*Issatchenkia* 13	*P. kudriavzevii/I. orientalis*	I II III	61824 2748402 2588444	106	106 108 107	99 96 97
*Issatchenkia* 14	*P. kudriavzevii/I. orientalis*	I II III	61824 2748402 2588444	106	106 108 107	99 96 97
*Issatchenkia* 15	*P. kudriavzevii/I. orientalis*	I II III	61824 2748402 2588444	107	107 108 107	100 98 99
*Issatchenkia* 16	*P. kudriavzevii/I. orientalis*	I II III	61824 2748402 2588444	106	106 108 107	100 97 98
*Issatchenkia* 17	*P. kudriavzevii/I. orientalis*	I II III	61824 2748402 2588444	106	106 108 107	99 96 97
*Issatchenkia* 18	*P. kudriavzevii/I. orientalis*	I II III	61824 2748402 2588444	106	106 108 107	99 96 97
*Issatchenkia* 19	*P. kudriavzevii/I. orientalis*	I II III	61824 2748402 2588444	106	106 108 107	99 96 97
*Issatchenkia* 20	P. kudriavzevii *	I II III	61824 2748402 2588444	106	106 108 107	96 94 94

*The second column shows the identification (≥97% sequence identity) of the ASVs based on sequence alignment against type strain sequences present in the non-redundant nucleotide (nt) database of the National Centre for Biotechnology Information (NCBI). If the best match was below 97% sequence identity, the sequence was compared to all sequences in the nt database and the result (≥97% sequence identity) is indicated with an asterisk. The other columns represent the alignment of the ASVs against the genome sequence of the starter culture strain Pichia kudriavzevii IMDO 020508, comprising its chromosome number, the position on the chromosome where the ASVs align, the length of the ASVs, the aligned length, and the sequence identity of the alignment.*

The alpha-diversity of the ASVs of the genera corresponding with the species of the inoculated strains was, in general, lower in those cocoa fermentation processes inoculated with the respective strains, indicating the efficacy of the starter culture addition (Table 5). The lowest diversity of the *Lactobacillus* and *Acetobacter* ASVs was found in the fermentation processes inoculated with *Liml. fermentum* IMDO 0611222 and *A. pasteurianus* IMDO 0506386. Oppositely, the diversity of the *Saccharomyces* ASVs was lower in the spontaneous cocoa fermentation processes, as *Saccharomyces* was only present at low relative abundances and at a few time points in these processes. Finally, the evenness of the *Issatchenkia* (*P. kudriavzevii*) ASVs was lower in those fermentation processes (AFSC I and II) inoculated with *P. kudriavzevii* IMDO 020508.

**TABLE 5:** Alpha-diversity metrics [Shannon (diversity) and Pielou (evenness) indexes] based on the relative abundances of the amplicon sequence variants (ASVs) belonging to the four genera to which the species of the inoculated strains belonged.

**Genus**	**Negative control**	**Positive control**	**AFSC I**	**AFSC II**
**Shannon**				
*Lactobacillus*	0.996	0.203	0.191	0.021
*Acetobacter*	1.504	0.154	0.659	0.331
*Saccharomyces*	0.166	0.371	0.772	0.590
*Issatchenkia*	1.059	0.696	0.709	0.715
**Pielou**				
*Lactobacillus*	0.907	0.093	0.098	0.013
*Acetobacter*	0.773	0.140	0.951	0.478
*Saccharomyces*	0.151	0.207	0.557	0.303
*Issatchenkia*	0.964	0.502	0.395	0.310

*The type of fermentation process is as explained in the legend of Figure 1.*

### Substrate Consumption and Metabolite Production Dynamics

#### Simple Carbohydrate Consumption

Differences in substrate consumption and metabolite production dynamics were found when comparing the spontaneous (NC) and starter culture-initiated cocoa fermentation processes examined (PC, AFSC I, and AFSC II) and in particular as a function of the starter culture mixture used (Supplementary Figure 2). A faster consumption of the glucose and fructose present in the cocoa pulp (*p* < 0.05) was found in the fermentation processes initiated with *S. cerevisiae* IMDO 050523 (PC and AFSC II), as reflected in their concentrations after 44 h of fermentation (Supplementary Figure 2). Whereas sucrose was only found at very low concentrations in the cocoa pulp, indicating that the cocoa pods used were mature, its high concentrations in the cocoa beans at the beginning of the fermentation processes (varying from 12.4 to 22.0 mg/g) halved after 92 h of fermentation (6.3–11.3 mg/g) (Supplementary Figure 3). The glucose and fructose concentrations ranged from 1.3 to 4.0 mg/g and from 1.0 to 4.0 mg/g, respectively, in the non-cured cocoa beans and decreased to concentrations of 0.7–2.2 mg/g and 0.4–1.4 mg/g by the end of the fermentation step. No significant differences (*p* > 0.05) were found in the simple carbohydrate concentrations in the cocoa beans during the fermentation step for the four different cocoa fermentation processes performed. After 2 days of drying, the concentrations of sucrose in the beans decreased on average with 62.2%, whereas glucose and fructose were found at higher concentrations (average increase of 76.4 and 113.5%, respectively) in the drying beans of all cocoa fermentation processes examined (Supplementary Figure 3). Afterward, the concentrations of these three carbohydrates increased as a function of the drying time, resulting in cured cocoa beans with concentrations of sucrose, glucose, and fructose varying from 2.8 to 11.6 mg/g, from 1.9 to 8.2 mg/g, and from 1.9 to 9.2 mg/g, respectively.

**FIGURE 5:**
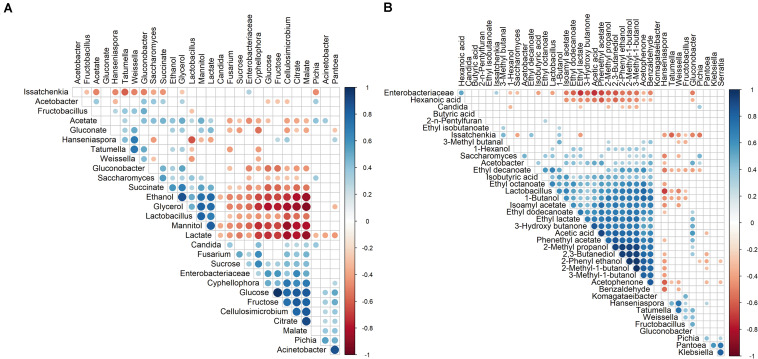
Spearman correlation analyses (*p* < 0.05) performed on the relative abundances (amplicon sequence variants) of the microbial genera and the concentrations of the substrates and metabolites **(A)** and volatile organic compounds (VOCs) **(B)** quantified in the cocoa pulp during the fermentation step of eight different Costa Rican cocoa fermentation processes. Positive correlations are depicted in blue circles and negative correlations in red ones. The size and color intensity of the circles represent the degree of correlation.

Spearman correlation analysis showed a negative correlation between the simple carbohydrates mentioned above and the concomitant metabolites produced (see below), namely acetate, ethanol, glycerol, lactate, mannitol, and succinate (Figure 5A). Furthermore, a negative correlation occurred between both monosaccharides (glucose and fructose) and, in decreasing order, the genera *Lactobacillus*, *Saccharomyces*, *Acetobacter*, and *Gluconobacter*, but no correlation occurred for the genera *Hanseniaspora* and *Issatchenkia* (*P. kudriavzevii*).

#### Sugar Alcohol Production

Glycerol was produced progressively in the pulp of all cocoa fermentation processes, reaching maximum concentrations of 2.2–2.8 mg/g (Supplementary Figure 2). The concentrations of mannitol in the cocoa pulp remained at low values, although on average higher (*p* = 0.07) in all starter culture-initiated fermentation processes (maximum concentrations of 0.5–6.5 mg/g), and especially in F5 (6.5 mg/g after 92 h of fermentation), compared to the spontaneous ones (NC; maximum concentrations of 0.2 and 0.1 mg/g in F1 and F2, respectively). In general, the dynamics of these two sugar alcohols were comparable for both cocoa pulp and beans, but occurred approximately 10-fold less in concentrations in the latter ones (maximum concentrations of 0.2–0.3 mg/g and 0.1–0.6 mg/g for glycerol and mannitol, respectively). The concentrations of both sugar alcohols remained constant or slightly increased during the drying step (Supplementary Figure 3).

Spearman correlation analysis showed a positive correlation between glycerol and mannitol with, in decreasing order, the presence of *Lactobacillus* and *Saccharomyces* (Figure 5A). Additionally, glycerol showed a positive correlation with *Gluconobacter*.

#### Ethanol, Acetate, and Acetoin Production

In concordance with the simple carbohydrate consumption profiles, the production of ethanol was enhanced (*p* = 0.34) in the PC (maximum concentrations of 12.9 and 27.3 mg/g in the cocoa pulp of F3 and F4, respectively) and AFSC II cocoa fermentation processes (maximum concentrations of 26.2 and 24.8 mg/g in the cocoa pulp of F7 and F8, respectively) (Supplementary Figure 2). Regarding acetate production, the concentrations present in the pulp at the end of the cocoa fermentation processes were significantly higher (*p* < 0.05) in the NC (5.3 and 11.0 mg/g in F1 and F2, respectively) and even more in the PC fermentation processes (22.3 and 11.0 mg/g in F3 and F4, respectively) compared to both AFSC ones (Supplementary Figure 2). An increase of the concentrations of ethanol and acetate in the pulp led to an increase in the beans too (Supplementary Figures 2, 3). These two volatile metabolites were not or at very low concentrations found during the drying step (Supplementary Figure 3). In contrast, acetoin (concentrations of 0.2–1.1 mg/g in cocoa beans dried for 7 days), which was not found during the fermentation step, was found during the drying step of all cocoa fermentation processes examined and its concentrations remained stable or slightly decreased.

Spearman correlation analysis indicated that ethanol showed a positive correlation with, in decreasing order, *Lactobacillus*, *Saccharomyces*, *Gluconobacter*, and *Tatumella* (Figure 5A). Alternatively, a positive correlation occurred between acetate and, in decreasing order, *Gluconobacter*, *Saccharomyces*, *Acetobacter*, *Lactobacillus*, *Pichia*, *Acinetobacter*, and *Tatumella*.

#### Organic Acid Production

Citrate (concentrations of 15.3–24.0 mg/g) was present in the unfermented cocoa pulp and it was consumed faster (*p* = 0.07) in all starter culture-initiated cocoa fermentation processes (concentrations below 1.3 mg/g after 44 h of fermentation) than in the spontaneous ones (concentrations of 7.0 and 5.3 mg/g in F1 and F2, respectively, after 44 h of fermentation). The concentrations of lactate in the cocoa pulp were significantly higher (*p* < 0.05) in all starter culture-initiated fermentation processes (maximum concentrations of 5.6–10.9 mg/g) than in the spontaneous ones (maximum concentrations of 2.7 and 3.0 mg/g in F1 and F2, respectively). Gluconate was mainly produced during the first 24 h of fermentation, at the lowest concentrations in the PC fermentation processes, and its concentrations in the cocoa pulp differed among the biological replicates, reaching the highest ones in F2 (13.8 mg/g), F5 (13.6 mg/g), F6 (29.1 mg/g), and F8 (10.9 mg/g) after 20–44 h of fermentation. Succinate was mainly produced during those fermentation processes inoculated with *S. cerevisiae* IMDO 050523 (*p* < 0.05; concentrations of 0.3, 1.4, 0.6, and 1.2 mg/g after 44 h of fermentation in F3, F4, F7, and F8, respectively). Malate (concentrations of 1.3–2.5 mg/g) was only present in the cocoa pulp at the beginning of the fermentation processes, being consumed to depletion after 20–44 h of fermentation. Citrate was found in non-cured cocoa beans (3.9–9.1 mg/g) and slightly decreased over the fermentation course (4.0–5.9 mg/g) in each cocoa fermentation process performed (Supplementary Figure 3). Apart from citrate, only the organic acids lactate and gluconate could be quantified in the cocoa beans, but their concentrations remained always below 1 mg/g. A slight increase of the lactate, gluconate, succinate, and malate concentrations was found in the cocoa beans during the drying step (Supplementary Figure 3). A much sharper increase occurred in the case of the citrate concentrations.

Spearman correlation analysis indicated a negative correlation between citrate and, in decreasing order, *Lactobacillus*, *Gluconobacter*, and *Saccharomyces*, and between malate and, in decreasing order, *Lactobacillus* and *Gluconobacter* (Figure 5A). Lactate was only positively correlated with *Lactobacillus* and gluconate with, in decreasing order, *Weissella* and *Tatumella*. Finally, succinate showed a positive correlation with, in decreasing order, *Saccharomyces*, *Gluconobacter*, and *Lactobacillus*.

#### Volatile Organic Compound Production

The HS/SPME-GC-TOF-MS screening of the VOC fractions of both cocoa pulp and beans of all cocoa fermentation processes (fermentation step) together revealed 86 different VOCs (84 different ones in the cocoa pulp and 65 different ones in the cocoa beans), encompassing 30.2% esters, 23.3% alcohols, 19.8% aldehydes, 11.6% ketones, 10.5% organic acids, 2.3% furans/furanones, 1.2% terpenes/terpenoids, and 1.2% phenolic compounds (Supplementary Table 1).

**FIGURE 6:**
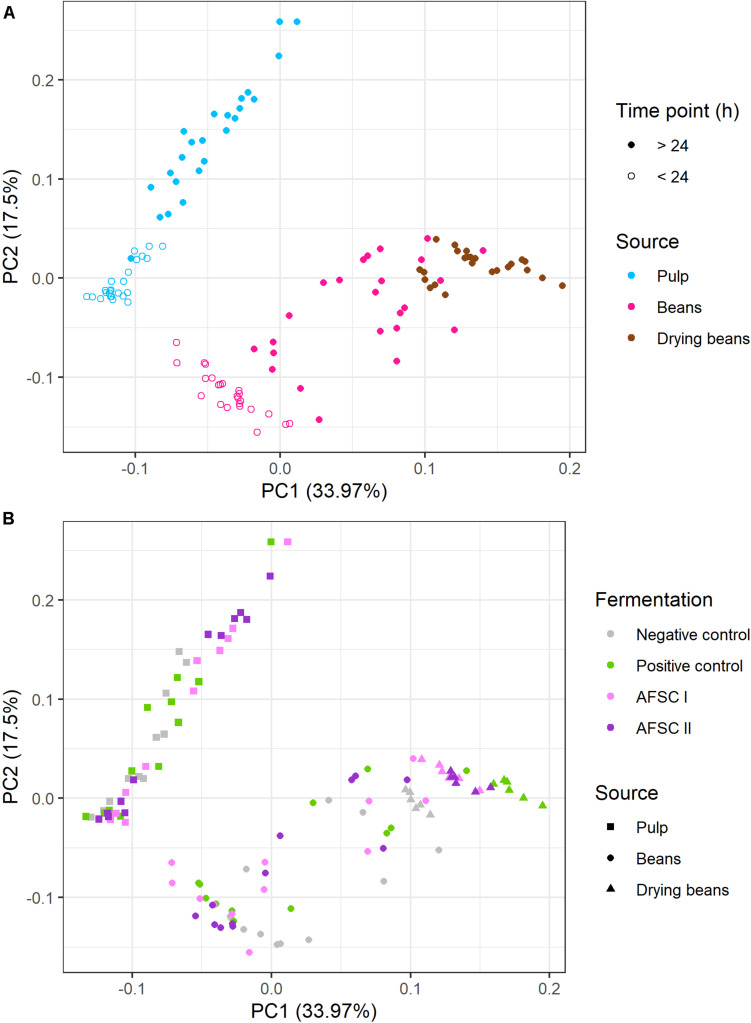
Principal component analysis performed on 29 volatile organic compounds (VOCs) quantified by liquid injection gas chromatography with tandem mass spectrometry (LI-GC-MS/MS) in the pulp and beans of the fermentation and drying steps of eight different Costa Rican cocoa bean curing processes. The type of fermentation process is as explained in the legend of Figure 1. **(A)** Influence of the source and fermentation duration, namely cocoa pulp (turquoise), beans (pink), and drying beans (brown), taken during the first 24 h of fermentation (open circles) and after 24 h of fermentation (closed circles). **(B)** Influence of the type of fermentation process, namely cocoa pulp (squares), beans (circles), and drying beans (triangles) withdrawn from the negative control (gray), positive control (green), and the adapted functional starter culture-initiated fermentation processes [AFSC I (pink) and AFSC II (violet)].

A total of 29 of these VOCs were quantified by LI-GC-MS/MS for each time point of the fermentation and drying steps for all cocoa bean curing processes (Supplementary Figures 4, 5). A PCA based on these VOC concentrations revealed two PCs, explaining more than 50% of the total variance, resulting in an influence of mainly the sample source and fermentation duration (Figure 6). PC1 was characterized by high positive loadings of 2-methyl-1-butanol, 3-methyl-1-butanol, 3-methyl butanal, isoamyl acetate, and phenylethyl acetate, which were more abundant in the cocoa beans. PC2 was characterized by high negative loadings of 2,3-butanediol and 2-phenyl ethanol, which were produced at higher concentrations in all starter culture-initiated fermentation processes. According to the source of the samples (pulp, beans, or drying beans), two separate clusters could be distinguished, reflecting the different VOC concentrations in the cocoa pulp and cocoa beans (Figure 6A). Yet, within the cocoa bean cluster, the drying beans clustered separately, reflecting the effect of the drying step on the VOC compositions of the cured cocoa beans. Also, as the VOC concentrations changed as a function of the fermentation time in both pulp and beans, two clusters representing fermentation before and after 24 h could be distinguished (Figure 6A). According to the type of cocoa fermentation processes performed (spontaneous or starter culture-initiated), also two clusters could be distinguished regarding the cocoa pulp (the PC and NC fermentation processes clustering together *versus* both AFSC fermentation processes; Figure 6B). For the cocoa beans, this clustering was less pronounced. However, the drying cocoa beans did cluster according to the starter culture used.

**FIGURE 7:**
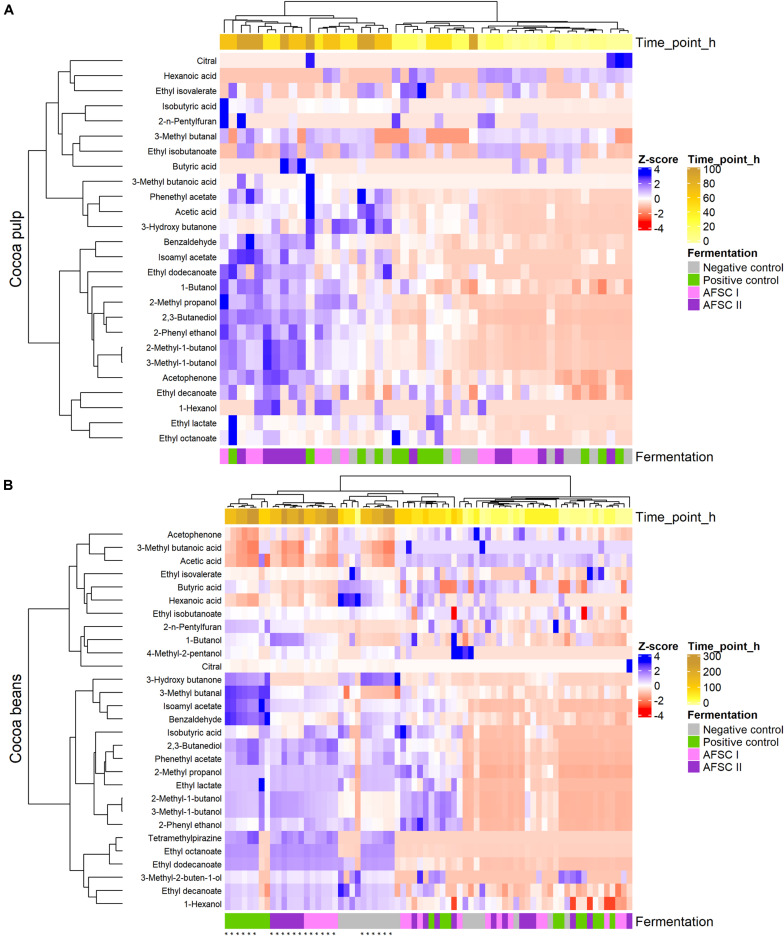
Hierarchical clustering analysis performed on 29 volatile organic compounds (VOCs) quantified by liquid injection gas chromatography with tandem mass spectrometry (LI-GC-MS/MS) in the pulp **(A)** and beans **(B)** of the fermentation and drying steps of eight different Costa Rican cocoa bean curing processes. The type of fermentation process is as explained in the legend of Figure 1. Concentrations were normalized and are represented as *Z*-scores to assess their shifts across the curing processes and unravel differences among these processes. The source of the samples (fermentation or drying step) is represented in the bottom row (drying samples are indicated with asterisks). The fermentation and drying duration of each sample is represented in the top yellow row.

Hierarchical clustering analysis of the heatmaps representing the concentrations of the 29 quantified VOCs of both cocoa pulp and beans mentioned above showed differences among the cocoa fermentation processes performed (Figure 7). In the cocoa pulp, the VOCs of both starter culture-initiated AFSC fermentation processes clustered together, as a result of a significantly higher (*p* < 0.05) production of 3-methyl-1-butanol, 2-methyl-1-butanol, 2-phenyl ethanol, 2,3-butanediol, 1-butanol, ethyl decanoate, and benzaldehyde than in the spontaneous fermentation processes NC. This increase of the concentrations of these VOCs occurred earlier in the AFSC II fermentation processes, indicating a positive contribution of *S. cerevisiae* IMDO 050523. Isoamyl acetate was also found in higher concentrations (*p* < 0.05) in the cocoa pulp of the starter culture-initiated fermentation processes (both PC and AFSCs) compared with the spontaneous ones. 2-Methyl propanol was quantified at significantly higher (*p* < 0.05) concentrations in the cocoa pulp of the AFSC I fermentation processes. In the (drying) cocoa beans, 3-methyl butanal, isoamyl acetate, and benzaldehyde were quantified at higher concentrations (*p* < 0.05) in the PC fermentation processes than in the other ones. Hexanoic acid was mainly quantified in the drying beans of the NC fermentation processes but disappeared progressively. In a similar way, a number of VOCs produced during the fermentation step disappeared during the drying step (e.g., acetophenone, 3-methyl butanoic acid, acetic acid, and butyric acid). Apart from those VOCs, a general but slight decrease in the concentrations of most VOCs was found during the drying step, except for 2,3-butanediol, ethyl octanoate, phenylethyl acetate, and tetramethylpyrazine (TMP), which all increased during this drying step. TMP was the only pyrazine found in the drying step and it was found in the drying beans of all fermentation processes carried out.

Spearman correlation analysis indicated a positive correlation of the main genera [in decreasing order, *Lactobacillus*, *Acetobacter*, *Saccharomyces*, *Gluconobacter*, and *Issatchenkia* (*P. kudriavzevii*)] with the VOCs present in the cocoa pulp (Figure 5B). Several VOCs shared a positive correlation with *Lactobacillus* and *Acetobacter* (13 compounds) or *Saccharomyces* (9 compounds). Only *Lactobacillus* was positively correlated with 1-butanol and 1-hexanol. *Issatchenkia* (*P. kudriavzevii*) was the only genus positively correlated with the presence of 3-methyl butanal.

## Discussion

The present study comprised a multiphasic approach of one spontaneous and three starter culture-initiated cocoa fermentation processes, thereby also assessing the influence of the yeast *P. kudriavzevii*. It compared selective enumeration, rRNA-PCR-DGGE community profiling of agar plate washes, and metagenetic analysis based on total cocoa pulp-bean mass DNA to characterize their microbial community dynamics and species diversities. It further assessed fine-scale (strain-level) growth monitoring of the starter cultures added by applying an ASV approach. Its metabolomics, including VOCs that are of sensory importance, targeted both pulp and beans during both the fermentation and drying steps of all cocoa bean curing processes examined.

A straightforward correlation was shown between the main microbial species found in each of the cocoa fermentation processes carried out and the course of pH, temperature, and metabolite compositions of both pulp and beans. First, a restricted bacterial diversity and a rather wide diversity of yeasts, the latter in particular during the spontaneous fermentation processes, occurred. Concerning the LAB communities, high relative abundances of *Weissella* in the spontaneous (NC) and *Lactobacillus* in all starter culture-initiated fermentation processes, the latter being inoculated with *Liml. fermentum* IMDO 0611222 (formerly classified as *Lb. fermentum*), occurred throughout fermentation. The prevalence of *Weissella* during cocoa fermentation processes is not common and typically reflects environmental contamination (Camu et al., 2007). Yet, *Weissella ghanensis*, *Weissella fabalis*, and *Weissella fabaria* were first isolated from Ghanaian/Brazilian cocoa heap/box fermentation processes (De Bruyne et al., 2008, 2010; Snauwaert et al., 2013). Concerning the yeast communities, *Hanseniaspora* was always present, followed by *S. cerevisiae* and *P. kudriavzevii*, the latter species only when it was inoculated. Further, the starter culture inoculation of the well-adapted yeast, LAB, and AAB strains could effectively control the microbial compositions of the fermenting cocoa pulp-bean mass, as shown by rRNA-PCR-DGGE community profiling (species level) and ASV analysis (strain level). Indeed, a deeper analysis of the ASVs allowed to follow the dynamics of different variants within species of the genera to which the inoculated strains belonged (i.e., *Lactobacillus*, *Acetobacter*, *Saccharomyces*, and *Pichia*) and, hence, evaluate the success of the starter culture addition in the cocoa fermentation processes examined, better than PCR-DGGE. This way, the inoculated strains could be differentiated from very closely related microbial individuals, for instance belonging to the background microbiota of the fermenting cocoa pulp-bean mass. Indeed, single dominating ASVs corresponded with the inoculated variants belonging to the *Lactobacillus*, *Acetobacter*, and *Saccharomyces* genera. In the case of the bacterial ASVs, these variants prevailed in all starter culture-initiated fermentation processes, which were inoculated with the *Liml. fermentum* IMDO 0611222 and *A. pasteurianus* IMDO 0506386 strains. The alpha-diversity of these variants within those genera was hence pulled down in all starter culture-initiated fermentation processes when compared to the spontaneous ones. However, given that only the V4 region of the 16S rRNA gene was sequenced, the occurrence and possible growth and contribution to the cocoa fermentation process of very closely related bacterial strains in the natural background could not be excluded, as the starter culture strains used were natural isolates from cocoa fermentation processes performed before, albeit with a different cocoa variety in a different region. To be able to differentiate all strains and be assured that the starter culture strains indeed prevailed over the background microbiota, a more thorough approach will be required, for instance using an ASV analysis based on the full-length 16S rRNA genes (Callahan et al., 2019), or via high-throughput whole-genome sequencing of many isolates collected from the cocoa fermentation processes. Concerning the *Saccharomyces* ASVs, also a unique variant was present at high relative abundances only in those fermentation processes inoculated with *S. cerevisiae* IMDO 050523 (PC and AFSC II). Furthermore, the *Saccharomyces* variant found at high relative abundances in fermenting cocoa pulp-bean mass that was not inoculated with this strain (NC and AFSC I) differed from the main ones found in the PC and AFSC II fermentation processes, showing that the strain inoculated could be differentiated from the environmental ones, although the same reticence needs to be made, as also here only a part of the rRNA transcribed unit was considered. In the case of the *Issatchenkia* (*P. kudriavzevii*) ASVs, two main variants co-occurred, in the same ratio of their relative abundances, during those fermentation processes inoculated with *P. kudriavzevii* IMDO 020508 (AFSC I and AFSC II). Indeed, as yeasts can have more than one copy of the rRNA transcribed unit in their genomes and those copy number variants can have higher mutation rates, different ITS1 sequences can be found in a single genome (Ganley and Kobayashi, 2007; Black et al., 2013; Steenwyk and Rokas, 2018). This was the case for *P. kudriavzevii* IMDO 020508, as its eight different ITS1 sequence variants were spread over 27 loci in the genome (consisting of five chromosomes). Interestingly, from these eight different ITS1 sequences of *P. kudriavzevii* IMDO 020508, only two were amplified by PCR in the case of the AFSC fermentation processes, and those were not found in the highest copy numbers in the genome of this strain. This indicated that there was no correlation between the number of copies of the rRNA transcribed units and the amplification of the ITS1 region through PCR.

Second, the microbial community dynamics showed, in general, a survival of the yeast populations upon 4 days of fermentation, in contrast with a usual decrease of the yeast counts after 48 h of fermentation during common cocoa fermentation processes (Ardhana and Fleet, 2003; Lagunes Gálvez et al., 2007; Camu et al., 2008; Papalexandratou et al., 2011a, 2013; Meersman et al., 2013). This could be ascribed to the limited increase in temperature of the fermenting cocoa pulp-bean mass, in turn indicating slow progressing fermentation processes. The yeast communities usually decrease at temperatures above 45°C, whereas the temperature of the fermentation processes of the present study never exceeded on average 37°C (Schwan and Wheals, 2004; Daniel et al., 2009; De Vuyst and Weckx, 2016; Maura et al., 2016). This was most probably due to the open lid of the fermentation vessels during the aerobic phase, without covering the fermenting cocoa pulp-bean mass with banana leaves, which usually keep the fermentation heat inside. Alternatively, a reduced flow of sweatings may have promoted yeast growth and limited air ingress. This, in turn, limited the growth of AAB, which were mainly *Acetobacter* species and to a lesser extent *Gluconobacter* species, hence leading to low acetate concentrations and thus less fermentation heat. Indeed, the lowest fermentation temperatures occurred in the AFSC fermentation processes that were characterized by low AAB counts. A limited temperature increase could, however, be favorable for a higher VOC production (Papalexandratou and Nielsen, 2016). Yet, cocoa fermentation processes during which yeast communities prevail across the whole process also commonly occur, especially when yeast starter cultures are added (Leal et al., 2008; Lefeber et al., 2012; Meersman et al., 2015; Visintin et al., 2017; Ho et al., 2018). However, several factors may be responsible for a yeast decline during common cocoa fermentation processes, such as the exhaustion of appropriate energy sources, too high ethanol and acetate concentrations, as well as killer toxin production (De Vuyst and Leroy, 2020). Indeed, the acetate concentrations, together with the increasing temperature profiles, of the cocoa fermentation processes of the present study correlated with a small decline of the yeast counts in the NC and PC fermentation processes. Further, the rRNA-PCR-DGGE community profiling and ASV analysis showed a shift in the yeast communities in both the spontaneous (NC) and starter culture-initiated fermentation processes from *Hanseniaspora* to *Saccharomyces*. This is commonly seen not only in spontaneous cocoa fermentation processes but also in spontaneous wine fermentation processes, reflecting the sensitivity of the former less fermentative yeast genus toward high ethanol concentrations and elevated temperatures (Ardhana and Fleet, 2003; Daniel et al., 2009; Papalexandratou et al., 2013; Jolly et al., 2014; Meersman et al., 2015; De Vuyst and Weckx, 2016).

Third, the consumption of simple carbohydrates accelerated through the use of starter cultures, especially in those fermentation processes inoculated with *S. cerevisiae* IMDO 050523 (PC and AFSC II). This feature is one of the most commonly found characteristics of cocoa fermentation processes inoculated with starter cultures containing the yeast species *S. cerevisiae* (Lefeber et al., 2012; Ramos et al., 2014; Batista et al., 2015; Sandhya et al., 2016; Moreira et al., 2017; Visintin et al., 2017; Ho et al., 2018). A fast simple carbohydrate consumption is accompanied with an enhanced production of ethanol, reflecting the high ethanol production capacity and tolerance of *S. cerevisiae* (Schwan, 1998; Daniel et al., 2009; Batista et al., 2015; Moreira et al., 2017). This enhanced production of ethanol by *S. cerevisiae* could explain the intermediate maximum temperature during fermentation processes inoculated with *S. cerevisiae* IMDO 050523 (PC and AFSC II). However, the consumption of glucose and fructose could not only be ascribed to yeast activity but also to the addition of the strictly heterofermentative *Liml. fermentum* IMDO 0611222, as reflected in the higher production of lactate and mannitol in all starter culture-initiated fermentation processes compared to the spontaneous ones. The absence of mannitol in the spontaneous fermentation processes was due to the prevalence of *Weissella*, which does not possess the enzyme mannitol dehydrogenase necessary to reduce fructose, in contrast with *Liml. fermentum* (Camu et al., 2007; Gänzle, 2015). High concentrations of mannitol and lactate and low concentrations of ethanol may be related to the low simple carbohydrate consumption by *P. kudriavzevii* in fermentation processes inoculated with the latter yeast species (AFSC I and AFSC II). This shared consumption of simple carbohydrates between yeasts and LAB may be responsible for a low competitiveness of the *Pichia* species and, hence, its retarded growth. Furthermore, the absence of a negative correlation between the occurrence of *Issatchenkia* and *Hanseniaspora* and the glucose and fructose concentrations may also suggest a diminished consumption of the main carbohydrates present in the pulp by these yeasts, compared to that of *Saccharomyces* and *Lactobacillus*.

Fourth, the pH course of the cocoa fermentation processes performed was influenced by the application of the starter cultures used, which was in accordance with different consumption rates of citrate, in particular when the citrate-positive *Liml. fermentum* IMDO 0611222 was inoculated, and thus explaining the slower pH increase during the spontaneous fermentation processes. Although LAB are responsible for citrate consumption during cocoa fermentation processes, also some yeasts (e.g., *P. kudriavzevii*, *Pichia fermentans*, and *S. cerevisiae*) have been associated with citrate assimilation (Schwan and Wheals, 2004; Jespersen et al., 2005; Daniel et al., 2009; Ho et al., 2014; Samagaci et al., 2016). Yet, it has been demonstrated that not all strains of yeast species retrieved from cocoa fermentation processes consume citrate, especially when simple carbohydrates are extensively available (Jespersen et al., 2005; Daniel et al., 2009). In contrast, mainly members of other microbial groups, in particular LAB (e.g., *Liml. fermentum*), are efficient citrate converters (Ardhana and Fleet, 2003; Camu et al., 2007; Lefeber et al., 2010, 2011; Papalexandratou et al., 2011a; Ouattara et al., 2017); in addition, enterobacteria (Papalexandratou et al., 2011a; Illeghems et al., 2015b) and maybe even AAB (R. Pelicaen, L. De Vuyst, D. Gonze, and S. Weckx, unpublished results) can consume citrate. Other organic acids than citrate also influenced the pH course of the cocoa fermentation processes carried out, as shown by the higher production of gluconate and lactate in the AFSC I fermentation processes, explaining its continuous decreasing pH in their middle part. Enterobacteria can be indicated as the main producers of gluconate from glucose through its oxidation by glucose dehydrogenase (Ramachandran et al., 2006; Papalexandratou et al., 2011b; Illeghems et al., 2015b). Yet, in the PC fermentation processes, gluconate was produced at low levels, which may indicate the presence of different enterobacterial species. The positive correlation of gluconate with *Tatumella* may support this hypothesis. Traditionally, enterobacteria have been associated with poor fermentation and the formation of off-flavors and biogenic amines (Pereira et al., 2012; Illeghems et al., 2015b). However, more recent studies may point toward a positive effect of enterobacteria during cocoa fermentation processes regarding simple carbohydrate consumption, citrate consumption, and pectin degradation (Ardhana and Fleet, 2003; Garcia-Armisen et al., 2010; Papalexandratou et al., 2011a; Illeghems et al., 2015b). Succinate was mainly produced in the fermentation processes harboring *S. cerevisiae* IMDO 050523, a trait that has been shown before during cocoa fermentation processes (Mota-Gutierrez et al., 2018; Figueroa-Hernández et al., 2019). However, other cocoa fermentation studies have reported on the production of this organic acid by *Pichia* (Ho et al., 2015) and LAB too (Camu et al., 2007). Finally, the prompt consumption of malate at early stages of the cocoa fermentation processes could be ascribed to malolactic fermentation by LAB, as it occurs in wine, cider, and lambic beer production processes (Swiegers et al., 2005; De Roos et al., 2018).

A pH increase at the end of cocoa fermentation processes can be ascribed to the disappearance of acetate, due to diffusion into the beans, evaporation, or overoxidation by AAB. Yet, the highest acetate concentrations in both pulp and beans were only found toward the end of the fermentation processes examined (in particular in the NC and PC ones), although *A. pasteurianus* was present since its inoculation in all starter culture-initiated fermentation processes. However, acetate also accumulated in the beans of both AFSC fermentation processes, suggesting other potential sources of the production of acetate in the pulp, such as heterolactic fermentation of glucose and fructose reduction by *Liml. fermentum* or even yeast metabolism, and maybe differences in its diffusion rate and extent (De Vuyst and Leroy, 2020). Nonetheless, the dynamics of the concentrations of ethanol, acetate, glycerol, and mannitol showed the same trends in the cocoa pulp and cocoa beans throughout fermentation in all cocoa fermentation processes examined. This was made possible by diffusion of these metabolites from the pulp into the beans. Whereas all these metabolites are directly or indirectly involved in redox balancing of the microorganisms involved, ethanol (mainly produced by the yeasts) and acetate (mainly produced by the AAB through cross-feeding on ethanol produced by the yeasts) are desirable metabolites for cocoa bean curing, as they avoid the growth of undesirable microorganisms in the pulp, facilitate killing of the embryo together with an increasing temperature in the beans, and hence indirectly contribute to color and flavor development in the beans through the activation of invertase, peptidases, glycosidases and polyphenol oxidase, which is determined by the pH decrease and temperature increase in the beans (De Vuyst and Weckx, 2016; Kongor et al., 2016; De Vuyst and Leroy, 2020; Santander Muñoz et al., 2020).

Fifth, the dynamics and concentrations of the VOCs in the cocoa pulp differed from those in the cocoa beans, which could be ascribed to production in either the pulp (microbial activities, such as higher aldehydes, higher alcohols, organic acids, and esters) or the beans (endogenous plant metabolism, such as certain ketones and terpenes) or diffusion from the pulp into the beans, mainly higher alcohols, organic acids, and esters (Rodriguez-Campos et al., 2011, 2012; Kadow et al., 2013; Ho et al., 2014, 2018; Sukha et al., 2014; Cevallos-Cevallos et al., 2018; Chetschik et al., 2018; Assi-Clair et al., 2019; Castro-Alayo et al., 2019; Mota-Gutierrez et al., 2019; Rottiers et al., 2019). Consequently, higher aldehydes, higher alcohols, and esters produced by yeasts and LAB were the main contributors to the cocoa flavor potential (chocolate, floral, and fruity notes), the concentrations of which were usually higher toward the end of the fermentation processes, in particular for the starter culture-initiated ones. Whereas desirable organic acids are mainly produced by LAB and AAB, in turn explaining the positive correlation between those microorganisms and the production of VOCs, the furan/furanone, ketone, phenolic, and terpene contents depend on the cocoa variety harvested (Kadow et al., 2013; Cevallos-Cevallos et al., 2018; Santander Muñoz et al., 2020).

Volatile organic compounds produced during the fermentation and/or drying steps play an important role in the final flavor characteristics of the cured cocoa beans and, hence, the chocolates produced from their roasted counterparts (Schwan and Wheals, 2004; Afoakwa et al., 2008; Saltini et al., 2013; Kongor et al., 2016; De Vuyst and Leroy, 2020; Santander Muñoz et al., 2020). The use of appropriate yeast starter cultures may influence that (Crafack et al., 2014; Ramos et al., 2014; Meersman et al., 2016; Ho et al., 2018; Mota-Gutierrez et al., 2018; Assi-Clair et al., 2019). Indeed, the application of the starter culture mixtures examined in the present study was reflected in the concentrations of the VOCs produced during the fermentation step, especially in the cocoa pulp. In particular, the yeast species inoculated played an important role, confirming earlier investigations (Lefeber et al., 2012; Meersman et al., 2015, 2016; Ho et al., 2018). Whereas yeasts are good producers of higher aldehydes, higher alcohols and esters, LAB also produce these compounds, except for esters (De Vuyst and Leroy, 2020). However, an indirect impact on ester formation either through the production of higher aldehydes and alcohols that are further metabolized by yeasts or the establishment of favorable fermentation conditions for VOC production might happen, further explaining the positive correlation between the presence of *Lactobacillus* (*in casu Liml. fermentum*) and the production of VOCs. Moreover, a greater contribution to the production of these compounds was obtained from the *S. cerevisiae* strain inoculated than from the *P. kudriavzevii* one. However, both AFSC fermentation processes inoculated with *P. kudriavzevii* IMDO 020508 showed a higher production of certain VOCs in the cocoa pulp, such as 3-methyl butanal, 2-phenyl ethanol and ethyl decanoate, which contribute to floral and fruity notes. Alternatively, the production of VOCs by *P. kudriavzevii* is strain-dependent (Pereira et al., 2017). Yet, the production of VOCs may also depend on different process factors, such as the fermentation method and cocoa variety, besides temperature and acidity (Kadow et al., 2013; Crafack et al., 2014; Sukha et al., 2014; Kongor et al., 2016; Meersman et al., 2016; Chetschik et al., 2018; Mota-Gutierrez et al., 2018; Castro-Alayo et al., 2019; Rottiers et al., 2019). Indeed, high temperatures and high acidity during the late stages of cocoa fermentation processes are desired for the production of VOCs in the cocoa pulp and their migration and retention in the beans (Kadow et al., 2013; Ho et al., 2014, 2018; Chetschik et al., 2018; Castro-Alayo et al., 2019). During the present study, the highest temperatures and acetate concentrations were obtained in the PC fermentation processes, in which the concentrations of certain higher alcohols and esters were better retained in the final cured cocoa beans. Consequently, the contribution of the fermentation microbiota on the final flavor of cured cocoa beans may be ascribed to their direct production of VOCs as well as their effects on the physicochemical changes during the fermentation and drying steps. Alternatively, the concentrations of VOCs may decrease (e.g., certain alcohols, ketones, and organic acids) or increase (e.g., certain simple carbohydrates, sugar alcohols, and organic acids) during the drying step. The latter may be linked to the loss of water from the beans upon drying. Whereas the loss of acetate is a desirable feature of the drying step, as remaining acetate in the beans upon drying can lead to unwanted acidity in the concomitant chocolates in the case of insufficient conching, the presence of high concentrations of reducing sugars in the dry cocoa beans is important for the development of cocoa flavor compounds through Maillard reactions with free amino acids during roasting (Afoakwa et al., 2008; Crafack et al., 2014; De Vuyst and Leroy, 2020). Further, during the drying step, VOCs are further developed (Afoakwa et al., 2008; Rodriguez-Campos et al., 2011). However, most of the VOCs quantified in the present study were produced during the fermentation step and slightly decreased during drying. Oppositely, TMP was only formed in drying cocoa beans, but not during fermentation. TMP is the most commonly found pyrazine in cocoa, contributing cocoa- and coffee-associated notes (Rottiers et al., 2019). It can be produced from acetoin, which was formed in the drying beans as well (and not during the fermentation step), probably non-enzymatically due to the proper environmental conditions, such as acidity and temperature (Xiao et al., 2014).

Finally, most studies on the impact of yeast starter cultures on flavor formation have been performed with Forastero cocoa, given its role in bulk chocolate production (intense cocoa flavor) and long fermentation time required (Rodriguez-Campos et al., 2011, 2012; Cevallos-Cevallos et al., 2018; Castro-Alayo et al., 2019). However, the application of appropriate yeasts as part of the starter cultures may also further accentuate the high flavor potential of shortly fermented Trinitario (and Criollo) cocoa, as shown during the present study.

## Conclusion

The application of a straightforward microbiological technique, namely ASV analysis, contributed to the follow-up of microbial strains inoculated in the cocoa pulp-bean mass during starter culture-initiated cocoa fermentation processes, in a more reliable and sensitive way than other commonly used techniques (DGGE, q-PCR, PFGE, or an OTU-based approach). The positive control (*S. cerevisiae* IMDO 050523, *Liml. fermentum* IMDO 0611222, and *A. pasteurianus* IMDO 0506386) fermentation processes reached a higher degree of fermentation than the spontaneous ones. In turn, the AFSC fermentation processes seemed to need 1 day more of fermentation to reach the same fermentation degree. In general, all starter culture-initiated cocoa fermentation processes showed an enhanced production of desired metabolites, in particular VOCs (in general higher aldehydes, higher alcohols, and esters and, in particular, 3-methyl butanal, 2-phenyl ethanol, and ethyl decanoate in the case of *P. kudriavzevii* and isoamyl acetate in the case of *S. cerevisiae*), resulting in richer cocoa bean VOC profiles than those resulting from the spontaneous fermentation processes.

## Data Availability Statement

The amplicon sequences are available under the accession numbers ERX4611870–ERX4611923 in the European Nucleotide Archive of the European Bioinformatics Institute (ENA/EBI).

## Author Contributions

AC and MV performed the field experiments and plating. CD-M performed the culture-independent, bioinformatic, and statistical analysis. CD-M and DVDV performed the metabolite analyses. CHA, SW, and LDV contributed to the coordination of the field experiments. SW and LDV designed and supervised the work. LDV was responsible for funding. CD-M drafted the manuscript. CD-M, DVDV, SW, and LDV revised the manuscript. CD-M and LDV edited the manuscript. All authors read and approved the final version of the manuscript.

## Conflict of Interest

The authors declare that the research was conducted in the absence of any commercial or financial relationships that could be construed as a potential conflict of interest.
