# Supplementary material for: Curing of Cocoa Beans: Fine-Scale Monitoring of the Starter Cultures Applied and Metabolomics of the Fermentation and Drying Steps
Source: Front Microbiol. 2021 Jan 11;11:616875. doi: 10.3389/fmicb.2020.616875 (PMC7829357; doi:10.3389/fmicb.2020.616875)
Supplement: Supplementary file 1 [file Data_Sheet_1.docx]

Supplementary Material

# SUPPLEMENTARY TABLES

**Table S1.** Volatile organic compounds (VOCs) detected through headspace/solid-phase microextraction coupled to gas chromatography with time-of-flight mass spectrometry (HS/SPME-GC-TOF-MS) in the cocoa pulp and cocoa beans of eight Costa Rican cocoa fermentation processes, whose set-up is explained in the legend of Figure 1. VOCs that were only detected in the cocoa pulp and those that were only detected in the cocoa beans are indicated with a single (*) or double asterisk (**), respectively (no asterisk means that those VOCs were detected in both pulp and beans). The flavor notes attributed to each of these compounds in relation to chocolate are indicated between brackets (Pubchem database).

| **Alcohols** | **Aldehydes** | **Ketones** | **Organic acids** | **Esters** | **Others** |
| --- | --- | --- | --- | --- | --- |
| [1-Butanol](http://www.flavornet.org/info/71-36-3.html) (fruity) | 2-cis-Heptenal* (dairy) | [2,3-Pentadione](http://www.flavornet.org/info/600-14-6.html) (creamy, caramel) | [Acetic acid](http://www.flavornet.org/info/64-19-7.html) (vinegar) | [Ethyl 2-ethylbutanoate](http://www.flavornet.org/info/7452-79-1.html) (NA) | **Furans** |
| [1-Decanol](http://www.flavornet.org/info/112-30-1.html) (fatty) | [2-Hexenal](http://www.flavornet.org/info/6728-26-3.html) (NA) | [2-Butanone](http://www.flavornet.org/info/78-93-3.html) (fruity) | [Diethyl succinate](http://www.flavornet.org/info/123-25-1.html) (winy) | [Ethyl 3-methyl butanoate](http://www.flavornet.org/info/108-64-5.html) (fruity) | 2-Methyl-furan (chocolate) |
| [1-Heptanol](http://www.flavornet.org/info/111-70-6.html)* (NA) | [2-Methylbutanal](http://www.flavornet.org/info/96-17-3.html) (cocoa) | [2-Heptanone](http://www.flavornet.org/info/110-43-0.html) (cheesy) | Butyric acid (cheesy) | [Ethyl acetate](http://www.flavornet.org/info/141-78-6.html) (brandy) | 2-Pentyl-furan (floral) |
| [1-Hexanol](http://www.flavornet.org/info/111-27-3.html) (grassy) | [2-Nonenal](http://www.flavornet.org/info/2463-53-8.html)* (paper) | [2-Pentanone](http://www.flavornet.org/info/107-87-9.html) (fruity) | [Isobutyric](http://www.flavornet.org/info/97-62-1.html) acid (cheesy) | [Ethyl benzoate](http://www.flavornet.org/info/93-89-0.html) (flowery) |  |
| [1-Nonanol](http://www.flavornet.org/info/143-08-8.html)* (floral) | [3-Methylbutanal](http://www.flavornet.org/info/590-86-3.html)* (chocolate) | 1-Hydroxy-2-propanone* (malty) | [Dodecanoic](http://www.flavornet.org/info/106-33-2.html) acid (coconut) | [Ethyl decanoate](http://www.flavornet.org/info/110-38-3.html)* (pear) | **Terpenes** |
| [1-Octanol](http://www.flavornet.org/info/111-87-5.html) (almond) | 2-trans-[Heptenal](http://www.flavornet.org/info/18829-55-5.html)* (almond) | [Acetophenone](http://www.flavornet.org/info/98-86-2.html) (almond) | [Propionic](http://www.flavornet.org/info/105-37-3.html) acid (pungent) | Ethyl heptanoate* (winy) | Limonene (lemon, orange) |
| [1-Pentanol](http://www.flavornet.org/info/71-41-0.html) (balsamic) | [Hexanal](http://www.flavornet.org/info/66-25-1.html) (green) | [Acetoin](http://www.flavornet.org/info/513-86-0.html) (creamy) | [Decanoic](http://www.flavornet.org/info/111-82-0.html) acid* (grassy) | [Ethyl hexadecanoate](http://www.flavornet.org/info/628-97-7.html) (waxy) |  |
| 1-Propanol (candy) | [Nonanal](http://www.flavornet.org/info/124-19-6.html)* (floral) | [Diacetyl](http://www.flavornet.org/info/431-03-8.html) (buttery) | Valeric acid (pungent) | [Ethyl hexanoate](http://www.flavornet.org/info/123-66-0.html) (fruity) | **Phenolics** |
| [2,3-Butanediol](http://www.flavornet.org/info/513-85-9.html) (creamy) | [Octanal](http://www.flavornet.org/info/124-13-0.html) (citrus) | [Nonanone](http://www.flavornet.org/info/821-55-6.html) (green) | 2-Methylbutyric acid (cheesy) | [Ethyl isohexanoate](http://www.flavornet.org/info/25415-67-2.html)* (fruity) | Phenol (phenolic) |
| [2-Ethylhexanol](http://www.flavornet.org/info/104-76-7.html)* (rose) | [Pentanal](http://www.flavornet.org/info/110-62-3.html)** (almond) | [Butyro-lactone*](http://www.flavornet.org/info/96-48-0.html) (cheesy) |  | [Ethyl lactate](http://www.flavornet.org/info/97-64-3.html) (fruity) |  |
| [2-Heptanol](http://www.flavornet.org/info/543-49-7.html) (mushroom) | [Phenyl ethanal](http://www.flavornet.org/info/122-78-1.html)* (berry) |  |  | [Ethyl octanoate](http://www.flavornet.org/info/106-32-1.html) (apricot) |  |
| [2-Methyl-1-butanol](http://www.flavornet.org/info/137-32-6.html) (winy) | Butanal (green) |  |  | [Ethyl phenylacetate](http://www.flavornet.org/info/101-97-3.html)* (honey) |  |
| [2-Pentanol](http://www.flavornet.org/info/6032-29-7.html) (green) | [Isobutyraldehyde](http://www.flavornet.org/info/78-84-2.html) (caramel) |  |  | [Ethyl propionate](http://www.flavornet.org/info/105-37-3.html) (strawberry) |  |
| [2-Phenyl ethanol](http://www.flavornet.org/info/60-12-8.html) (rose) | Methional (cooked potato) |  |  | [Hexyl acetate](http://www.flavornet.org/info/142-92-7.html)* (herb) |  |
| 3-Methyl-1-butanol* (malty) | Pentenal* (NA) |  |  | [Isoamyl acetate](http://www.flavornet.org/info/123-92-2.html)* (banana) |  |
| 4-Methyl-2-pentanol* (NA) |  |  |  | [Isobutyl acetate](http://www.flavornet.org/info/110-19-0.html) (herb) |  |
| [Benzyl alcohol](http://www.flavornet.org/info/100-51-6.html) (boiled cherries) |  |  |  | [Linalyl acetate](http://www.flavornet.org/info/115-95-7.html) (fruity) |  |
| [Ethanol](http://www.flavornet.org/info/64-17-5.html) (alcoholic) |  |  |  | [Methyl decanoate](http://www.flavornet.org/info/110-42-9.html) (winy) |  |
| [Isobutanol](http://www.flavornet.org/info/78-83-1.html) (winy) |  |  |  | [Methyl propanoate](http://www.flavornet.org/info/547-63-7.html) (fruity) |  |
| 2-Nonanol (cucumber) |  |  |  | β-Phenethyl acetate (NA) |  |
| Octanol (bitter almond) |  |  |  | Butyl acetate (glue) |  |
|  |  |  |  | Ethyl isobutyrate (fruity) |  |
|  |  |  |  | Heptyl acetate (floral) |  |
|  |  |  |  | Ethyl 3-hydroxy butanoate (marshmallow) |  |

NA, not available.


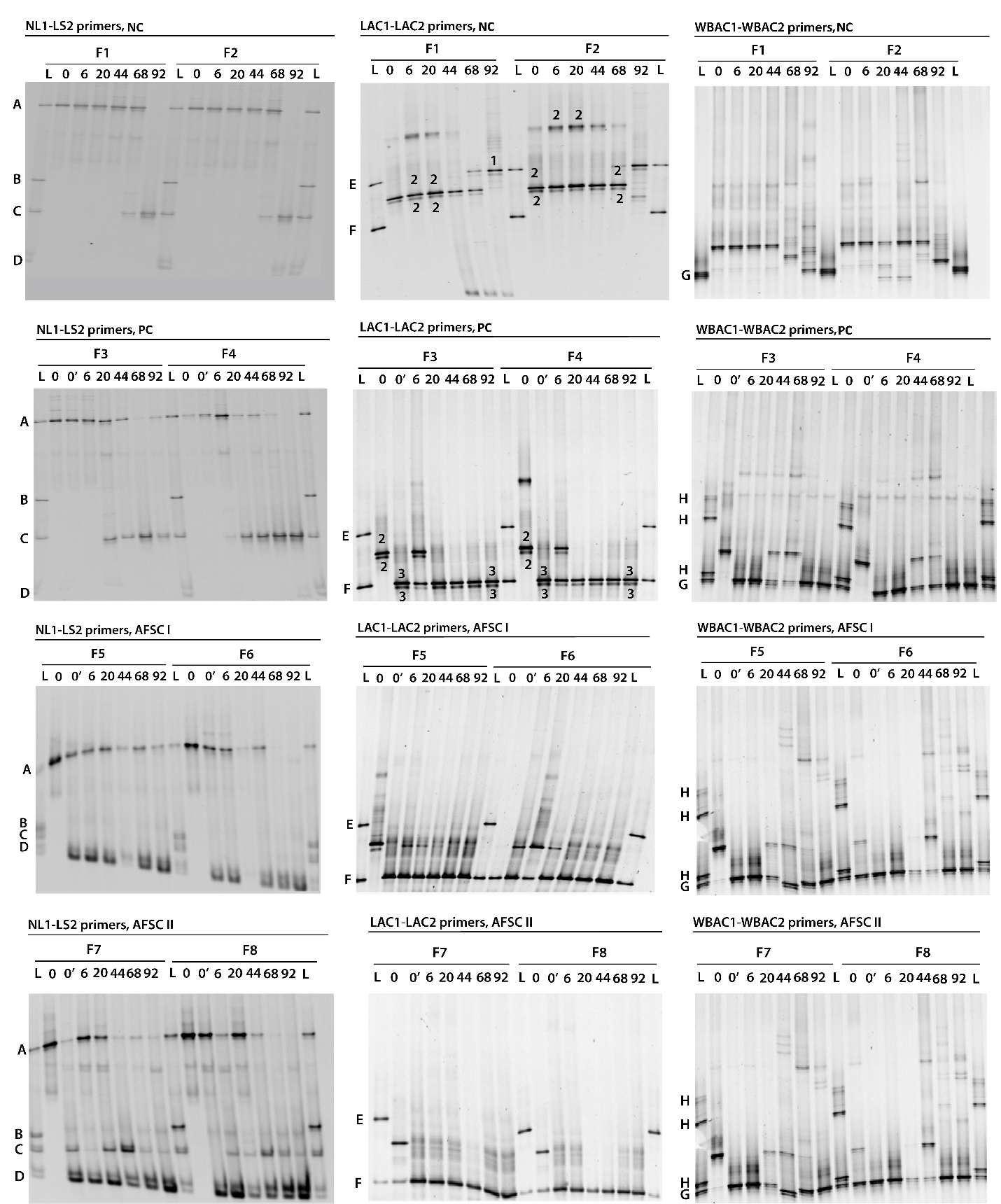


**Figure S1.** PCR-DGGE community profiles of agar plate washes, based on amplification of total DNA with the NL1-LS2 (yeasts; 35-60% denaturing gradient; left), LAC1-LAC2 (lactic acid bacteria; 35-60% denaturing gradient; middle) and WBAC1-WBAC2 (acetic acid bacteria; 45-70% denaturing gradient, right) primers, of 92 h Costa Rican cocoa fermentation processes carried out in eight different vessels, whose set-up is explained in the legend of Figure 1. The lane numbers represent the samples of different fermentation duration (h). The reference ladders **(L)** consisted of PCR amplicons obtained by using the respective primers with genomic DNA from pure cultures of **(A)** *Hanseniaspora opuntiae* IMDO 040108, **(B)** *Kluyveromyces marxianus* Y40, **(C)** *Saccharomyces cerevisiae* IMDO 050523, **(D)** *Pichia kudriavzevii* IMDO 020508, **(E)** *Lactiplantibacillus plantarum* IMDO 03P80, **(F** and **H)** *Limosilactobacillus fermentum* IMDO 0611222, and **(G)** *Acetobacter pasteurianus* IMDO 0506386. The closest relatives to the fragments sequenced were: **(1)** *Lactobacillus pentosus/plantarum/paraplantarum* (100 %; Genbank Accession no. CP032757.1, CP032751.1, CP032744.1; reclassified as *Lactiplantibacillus pentosus/plantarum/paraplantarum*), **(2)** *Weissella fabalis/beninensis/ghanensis* (100 %; Genbank Accession nos. NR_108858.1, NR_116326.1, NR_042663.1), and **(3)** *Lactobacillus fermentum* (100 %; Genbank Accession no. CP039750.1; reclassified as *Limosilactobacillus fermentum*).


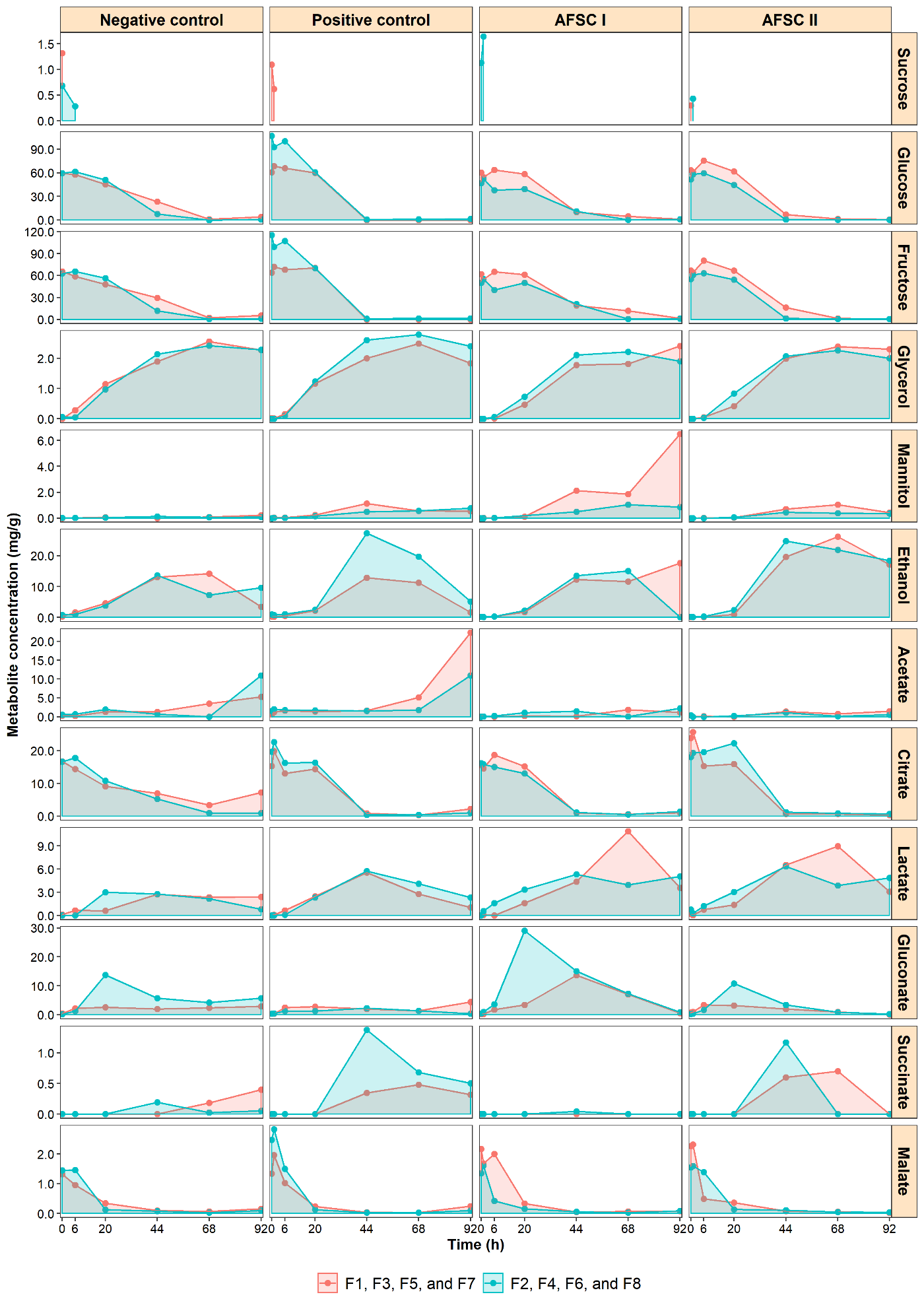


**Figure S2.** Dynamics of the concentrations of simple carbohydrates (sucrose, glucose, and fructose), sugar alcohols (glycerol and mannitol), ethanol, acetate, and other organic acids (citrate, lactate, gluconate, succinate, and malate) in the pulp during 92 h Costa Rican cocoa fermentation processes carried out in eight different vessels. The type of fermentation process (F1-F8) and sampling are as explained in the legend of Figure 1.


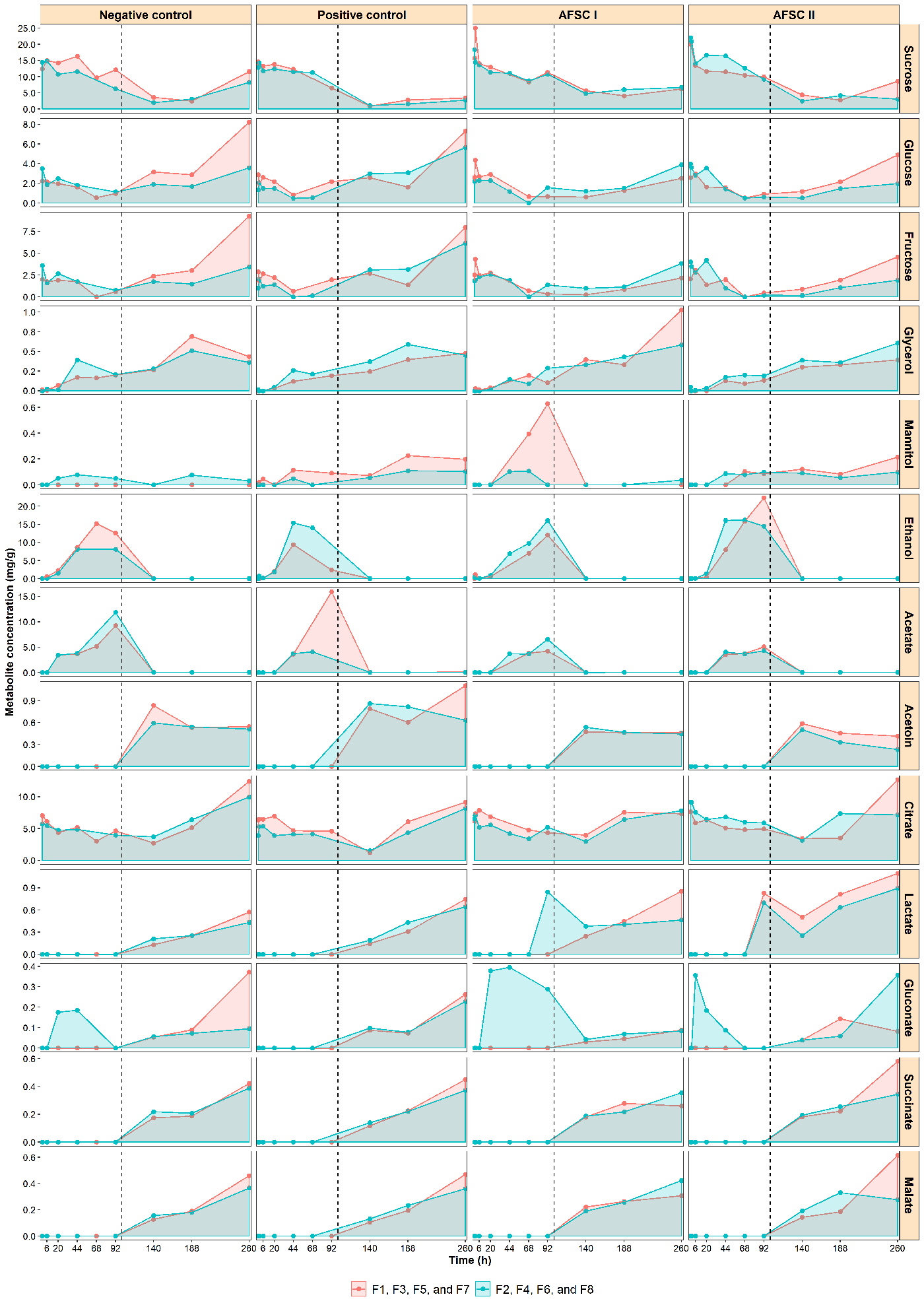


**Figure S3.** Dynamics of the concentrations of simple carbohydrates (sucrose, glucose, and fructose), sugar alcohols (glycerol and mannitol), ethanol, acetate, acetoin, and other organic acids (citrate, lactate, gluconate, succinate, and malate) in the beans during 92 h Costa Rican cocoa fermentation processes carried out in eight different vessels, followed by seven days of drying. The type of fermentation process (F1-F8) and sampling are as explained in the legend of Figure 1. The vertical dashed lines represent the transition from the fermentation step to the drying one.


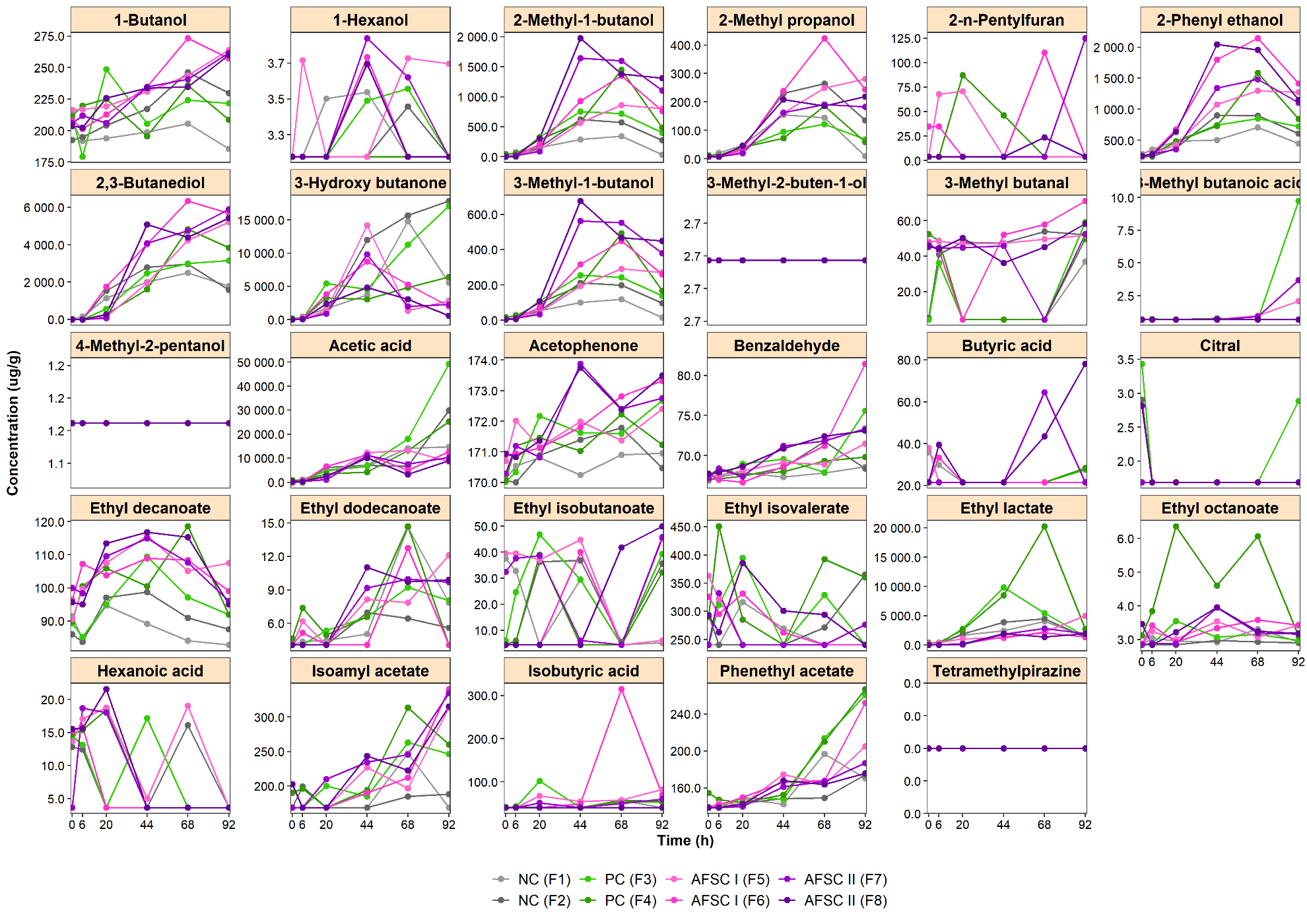


**Figure S4.** Dynamics of the concentrations of 29 volatile organic compounds (VOCs), as determined by liquid injection gas chromatography with tandem mass spectrometry (LI-GC-MS/MS), in the cocoa pulp during 92 h Costa Rican cocoa fermentation processes carried out in eight different vessels. The type of fermentation process (F1-F8) and sampling are as explained in the legend of Figure 1.


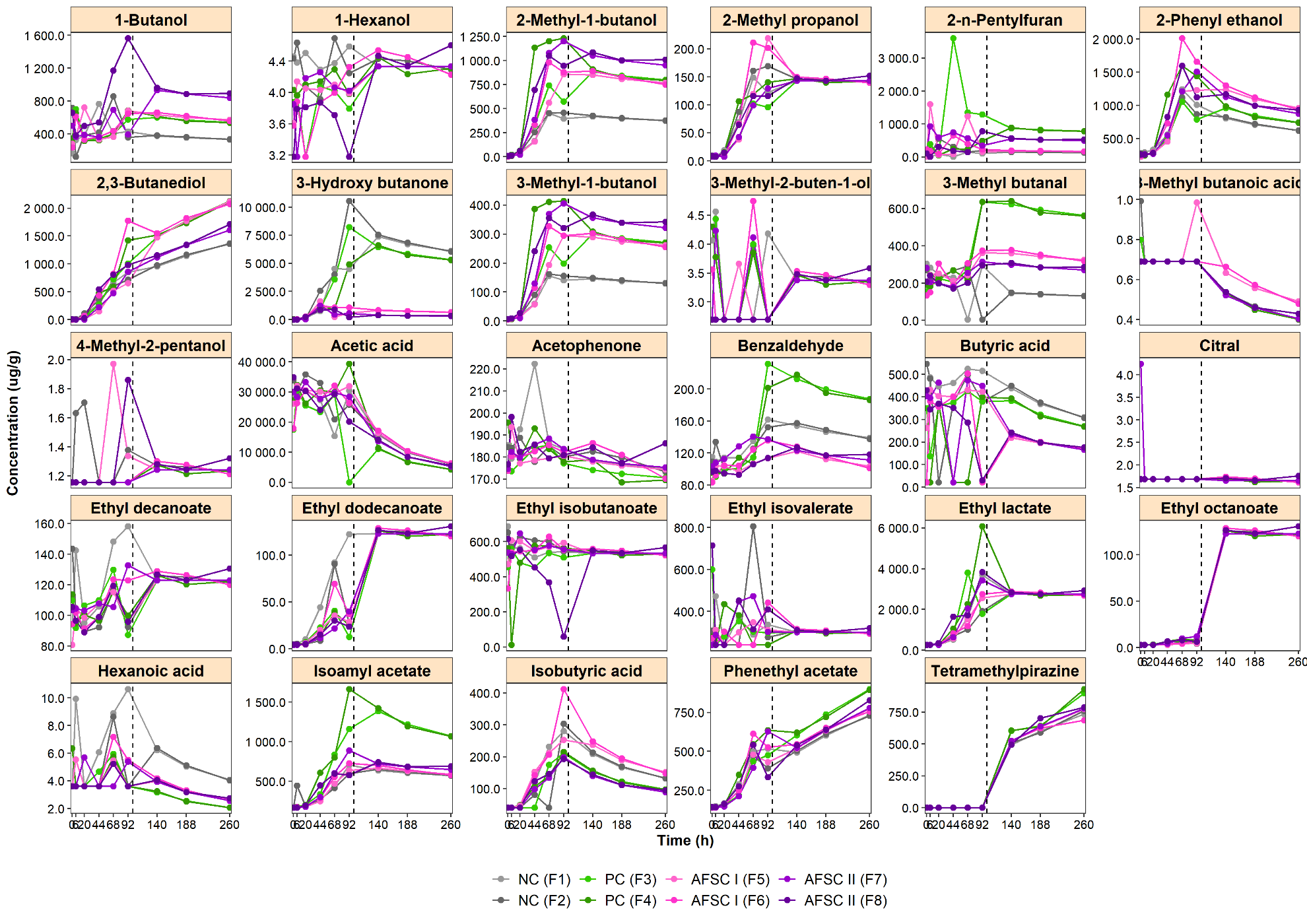


**Figure S5.** Dynamics of the concentrations of 29 volatile organic compounds (VOCs), as determined by liquid injection gas chromatography with tandem mass spectrometry (LI-GC-MS/MS), in the cocoa beans during 92 h Costa Rican cocoa fermentation processes carried out in eight different vessels, followed by seven days of drying. The type of fermentation process (F1-F8) and sampling are as explained in the legend of Figure 1. The vertical dashed lines represent the transition from the fermentation step to the drying one.
